# Evaluating the performance of existing tools to predict clinically significant prostate cancer in men with indeterminate lesions on biparametric MRI and development of a novel multiplex model: a prospective cohort study

**DOI:** 10.1016/j.eclinm.2025.103191

**Published:** 2025-04-03

**Authors:** Ahmad Abbadi, Martin Eklund, Anna Lantz, Andrea Discacciati, Lars Björnebo, Thorgerdur Palsdottir, Jan Chandra Engel, Fredrik Jäderling, Ugo Falagario, Henrik Grönberg, Tobias Nordström

**Affiliations:** a Department of Medical Epidemiology and Biostatistics, Karolinska Institutet, Solna, Sweden; b Department of Molecular Medicine and Surgery (Solna), Karolinska Institutet, Stockholm, Sweden; c Department of Clinical Sciences at Danderyds Hospital, Karolinska Institutet, Solna, Sweden; d Department of Radiology, Capio S:t Görans Hospital, Stockholm, Sweden; e Department of Urology and Kidney Transplantation, University of Foggia, Foggia, Italy; f Department of Oncology, Capio S:t Görans Hospital, Stockholm, Sweden

**Keywords:** Clinical decision-making, Magnetic resonance imaging, Prostate cancer, Cancer screening, Biomarkers

## Abstract

**Background:**

Indeterminate lesions on prostate MRI, such as PI-RADS 3, present a clinical challenge due to their equivocal nature, complicating biopsy decisions in men undergoing testing for prostate cancer. Understanding the predictive capacity of biomarkers and risk calculators is critical to improve clinical decision-making and reduce unnecessary biopsies.

**Methods:**

In this prospective cohort study, men with PI-RADS 3 findings on biparametric MRI (bp-MRI) who underwent combined biopsy (fusion targeted and systematic) in the STHLM3-MRI randomised clinical trial (first- and second-rounds) and at Capio St Göran's Hospital (Capio PCC), Sweden were included, representing screening-by-invitation, repeat screening, and clinical practice cohorts, respectively. Data collection occurred between Feb 5th, 2018, and Mar 4th, 2020, for STHLM3-MRI first-round screening, between Nov 10th, 2021, and Feb 20th, 2023 for second-round screening, and between Jan 7th, 2017 and June 30th, 2023 for Capio PCC. The data was collected directly from the participating laboratories using standardized reporting forms, medical charts, and additional study-specific data collection forms filled by patients. The primary outcome was detection of clinically significant prostate cancer (csPCa; ISUP ≥2) in men with PSA ≥3 ng/mL confirmed by the combined biopsy. The predictive capacity of the evaluated biomarkers (PSA density, the Stockholm3 test, prostate volume, MRI lesion volume ratio, and Stockholm3 density), as well as seven risk calculators, was assessed via the area under the curve (AUC) computed using logistic regression. Sensitivity and specificity of detecting csPCa and high-grade prostate cancer (ISUP ≥3) were reported. Complete-case analysis was performed for men with complete data on their PSA, prostate volume, Stockholm3 test, MRI lesion volume, findings on the digital rectal examination, family history of prostate cancer, and previous biopsy. The findings were contrasted to the analysis from the imputed dataset.

**Findings:**

Of the 6554 men included into the three cohorts, 1187 received PI-RADS score of 3 on the bp-MRI, and 1146 underwent combined biopsy. Of them, 900 had PSA ≥3 ng/mL, and 656 men were included in the complete-case analysis (169 from STHLM3-MRI first-round, 72 from the second-round, and 415 from Capio PCC). Overall, 370/900 men (41%) and 258/656 men (39%) had ISUP ≥2, but only 75/900 (8%) and 50/656 (8%) had ISUP ≥3. PSA density, tested risk calculators, and probability tests had low-to-moderate AUC (range 0.50–0.73; PSA density range 0.58–0.66, Stockholm3 range 0.59–0.67, lesion volume ratio range 0.54–0.63), and performed similarly across individual cohorts and the combined dataset in the complete-case and imputed dataset analysis. For detection of ISUP ≥2 based on STHLM3-MRI first-round, PSA density at 0.10 had a sensitivity of 69% (56%, 80%), specificity of 49% (39%, 58%), and missing 27% (6%, 61%) of ISUP ≥3, while a PSA density of 0.15 had a sensitivity of 37% (25%, 50%), specificity of 84% (76%, 90%), missing 45% (17%, 70%) of ISUP ≥3. The best-performing model based on STHLM3-MRI included age, prostate volume, Stockholm3 density and MRI lesion ratio, and reduced prostate biopsies by 33% (26%, 40%) while maintaining 98% (91%, 100%) sensitivity to detect ISUP ≥2 cancer, specificity of 50% (41%, 60%) and AUC of 0.82 (0.76, 0.87). Meanwhile, the best-performing model based on the complete-case combined dataset included age, prostate volume, PSA density, and Stockholm3 density, and reduce prostate biopsies by 26% (23%, 30%) with a sensitivity of 90% (85%, 93%), specificity of 36% (31%, 41%), and AUC of 0.70 (0.66, 0.74).

**Interpretation:**

Current risk-stratification tools and individual biomarkers perform suboptimally for guiding biopsy decisions in men with PI-RADS 3 lesions. The findings highlight the limitations of relying on PSA density alone and emphasize the need for caution in clinical recommendations. However, multiplex models might offer possibility to reduce unnecessary biopsies while maintaining high sensitivity for clinically significant prostate cancer detection. These findings should be externally validated and evaluated for cost-effectiveness.

**Funding:**

STHLM3-MRI clinical trial is funded by the 10.13039/501100002794Swedish Cancer Society (Cancerfonden), the 10.13039/501100004359Swedish Research Council (Vetenskapsrådet), the 10.13039/501100004359Swedish Research Council for Health Working Life and Welfare (FORTE), the Strategic Research Programme on Cancer (StratCan), Hagstrandska Minnesfonden, Region Stockholm, Svenska Druidorden, Åke Wibergs Stiftelse, the 10.13039/100017156Swedish e-Science Research Centre, the 10.13039/501100004047Karolinska Institutet, and Prostatacancerförbundet.

**Research in context**

**Evidence before this study**

We conducted a literature review of studies evaluating the predictive performance of biomarkers and risk calculators for clinically significant prostate cancer (csPCa) detection in men with equivocal PI-RADS 3 lesions on MRI. Searches were conducted across PubMed, Embase, and Web of Science up to November 30, 2024, using terms such as “PI-RADS 3,” “prostate cancer biomarkers”, “PSA density”, “risk calculators”, and “Stockholm3”. The evidence was limited by the use of single-centre data, heterogeneous populations, and variations in PI-RADS versions. Although biomarkers like PSA density have been studied previously, their performance in men with PI-RADS 3 lesions remained suboptimal, with AUCs often below 0.70. Risk calculators showed moderate predictive capacity, but their utility in men with PI-RADS 3 lesions was not specifically validated. Furthermore, many studies lacked external validation, while inter-observer variability in radiologist scoring further constrained reproducibility.

**Added value of this study**

Among men with equivocal lesions from the STHLM3-MRI trial and clinical practice cohort, individual biomarkers such as PSA density, Stockholm3, lesion volume ratio, and risk calculators demonstrated low-to-moderate predictive accuracy for detecting clinically significant prostate cancer. PSA density at varying clinically described thresholds would miss a significant number of the high-grade and clinically significant prostate cancers. When combining biomarkers in predictive models, the predictive capacity improved. The best-performing multiplex models when optimized for a clinically applicable cutoff point would exhibit high sensitivity, and reasonable specificity, which can aid in reducing benign and low-grade biopsies.

**Implications of all the available evidence**

Recommended biomarkers and tools perform suboptimally for biopsy decisions in men with equivocal lesions. However, when using multiple biomarkers optimized for men with equivocal lesions on MRI, the multiplex models could reduce the number of unnecessary biopsies performed while capturing the clinically significant prostate cancer cases. Caution is warranted when using PSA density in silo to guide biopsy decisions. Main limitation is the exploratory nature of the new multiplex models in this study, which require validation of the findings.

## Introduction

The usage of magnetic resonance imaging (MRI) reduces the number of unnecessary prostatic biopsies in men, thereby mitigating potential overdiagnosis.^1^^,^^2^ The scores are reported based on the Prostate Imaging-Reporting and Data System (PI-RADS).^3^^,^^4^

Lesions with a PI-RADS score of 3 pose a clinical challenge due to their equivocal nature.^3^ PI-RADS 3 prevalence ranged between 9 and 46% among performed MRIs on wide-range of centres and population groups (e.g., first biopsy, re-biopsy, etc.),^5^ with csPCa prevalence ranging on average from 2% to 38%, reaching up to 46% reported PCa detection based on retrospective and prospective studies using multiparametric MRIs.^6^, ^7^, ^8^ Although less than half of the patients with equivocal lesions have csPCa, the cancer prevalence remains clinically considerable, necessitating effective tools to reduce the number of unnecessary biopsies.

Various tools and biomarkers have been described to aid in the clinical decision-making process to biopsy patients with equivocal lesions. Prostate specific antigen (PSA) density (PSAD) is a notable biomarker, currently recommended in medical guidelines to inform biopsy decisions in men with PI-RADS 3,^9^^,^^10^ with thresholds of 0.10, 0.15, and 0.20 reflecting different risk groups.^11^, ^12^, ^13^ Additionally, numerous risk calculators have been published and described.^14^ Although Stockholm3 test has not been verified for its performance in men with PI-RADS 3 to inform biopsy decisions, it demonstrated high predictability to detect csPCa.^1^

Despite these advancements, there remain limitations to the existing evidence. Firstly, several studies relied on single centre-based populations.^11^^,^^12^^,^^14^ Secondly, the models were developed to detect csPCa in all screened men, and not specific to each PI-RADS score.^11^^,^^12^^,^^14^ In turn, the predictive capacity might not be optimal for men with equivocal lesions.^15^ Thirdly, the internal validity could be affected by potentially present selection bias due to centre-based designs or changing PI-RADS criteria version without reclassification and external validation by a single experienced radiologist.^13^^,^^15^ Fourthly, it is affected by inter-observer variability due to the level of knowledge and experience (both in terms of years and number of cases evaluated) by the radiologist.^16^^,^^17^

Henceforth, in this study, we aimed primarily to examine the predictive capacity of different biomarkers and risk calculators in men with PI-RADS score 3 to inform clinical decisions regarding prostate biopsies. As secondary objectives, we aimed to explore the predictive capacity of Stockholm3 test and ratio of biomarkers to prostate volume, and study if the performance of the biomarkers and risk calculators in men screened using PSA ≥3 ng/mL (primary population) differs from men screened with Stockholm3 as a reflex test following PSA testing.

## Methods

### Study population

**Fig. 1:**
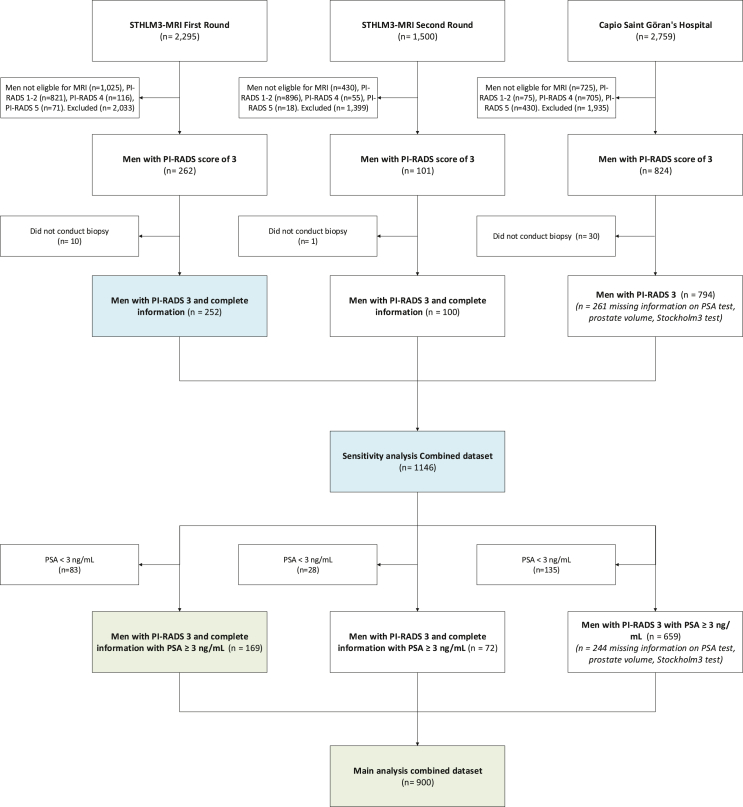
Flowchart of inclusion into the study. Description: Green, main analysis; blue, sensitivity analysis; MRI, Magnetic resonance imaging; PSA, prostate specific antigen; PI-RADS, Prostate Imaging-Reporting and Data System.

This study utilized three datasets: the first- and second-rounds of the STHLM3-MRI clinical trial,^1^^,^^18^ and data from Capio S:t Göran's Hospital's Prostate Cancer Centre (Capio PCC), a comprehensive referral urology clinic located in Stockholm, Sweden.^19^^,^^20^ The first-round of STHLM3-MRI represents a screening-by-invitation study cohort, the second-round represents repeated screening study cohort, and the Capio PCC represents referral hospital clinical-practice study cohort. In both the clinical trial and Capio PCC, we included consecutive men undergoing biparametric MRI (bpMRI) for early detection of prostate cancer based on a modified PI-RADS version 2.1 substituting multiparametric MRI with bpMRI.^21^ In the diagnostic chain of all three study cohorts, men had a PSA test, then a Stockholm3 test if PSA≥1.5 ng/mL, a short protocol (16 min) bpMRI if having elevated risk on the blood-sample (PSA ≥3 ng/mL or PSA 1.5–3 and Stockholm3 test ≥11%), and targeted plus systematic biopsies if having a PI-RADS score of 3 or higher (validated by single radiologist with more than 7-years’ experience in prostate MRI since the initiation of the studies). Further information on the external validation of the MRIs is available in the Supplementary File. Inclusion criteria for this study were men with a PI-RADS score of 3 who had PSA and Stockholm3 tests before MRI, underwent biopsy following their PI-RADS score, and had complete data on age, and Gleason grade. Those not meeting all inclusion criteria were excluded. Fig. 1 illustrates the study population flowchart for main and sensitivity analysis.

STHLM3-MRI first-round screening was conducted between Feb 5th, 2018, and Mar 4th, 2020, while second-round screening was conducted between Nov 10th, 2021, and Feb 20th, 2023. Data from Capio PCC was collected from Jan 7th, 2017 to June 30th, 2023.

Detailed information on the three cohorts used in this study, including the clinical, MRI, biopsy, and pathology procedures are available in the Supplementary File. Further information about the procedures and the full protocol are available elsewhere.^1^^,^^18^^,^^20^, ^21^, ^22^

### Ethical considerations

The clinical trial was approved by the Swedish Ethics Review Board (Dnr 2017/1280-31, 2018/2486-32, 2020-00014, 2020-00779, and 2021-03132), and the trial was registered on clinicaltrials.gov with ID NCT03377881.^23^ Capio Saint Görans Hospital data collection was approved by the Swedish Ethics Review Board (Dnr 2021-04395). To assure abidance with data management and safety, an independent Data Safety and Monitoring Board supervised the data management procedures related to STHLM3-MRI.^1^ Participants in the clinical trial were invited using mail invitations, and eligibility was assessed using a web-secure portal. All participants separately consented to participate in both the first- and second-round screenings. The participants in Capio Saint Görans Hospital did not require additional written consent as the data collected was part of the standard of care at the hospital. All clinicians who performed the biopsies, MRIs, and pathology assessments were highly experienced. Moreover, the study abided by the ethical standards in Helsinki Declaration and the regulations of the General Data Protection Regulation (GDPR).

### Outcomes

The primary outcome was clinically significant prostate cancer (csPCa), confirmed by combined biopsy (fusion targeted and systematic biopsies). Although the exact definition of csPCa is under consideration and pending international consensus, for the sake of the study it was defined as cases with Gleason score of ≥3 + 4 (ISUP grade group ≥2). High-grade prostate cancer (defined as Gleason score ≥4 + 3; ISUP grade group ≥3) was considered as an additional definition of csPCa. Clinically insignificant prostate cancer (defined as Gleason 3 + 3; ISUP grade group 1), and benign lesions (labelled as ISUP grade group 0) were further evaluated as secondary outcomes.

### Biomarker consideration

Biomarkers and cutoff points were selected using data-driven and literature-based approaches. PSAD,^12^ Stockholm3 test,^1^ prostate volume,^24^ age,^25^ digital rectal examination,^10^ and MRI lesion volume (LV)^26^ were considered based on previous studies that described their relation to biopsy outcomes. Additional novel variables were considered such as calculating Stockholm3 density (Stockholm3D) by dividing Stockholm3 test score by prostate volume.

MRI lesion volume ratio (LR) representing the proportion of the prostate the suspicious lesion occupies was computed by dividing the MRI lesion volume based on the largest suspicious lesion that led to the PI-RADS 3 scoring, and assumed to be ellipsoid, by prostate volume.

$$Lesion\;volume=\frac{\pi }{6}\;x\;Lesion\;Length\;x\;Lesion\;Width\;x\;Lesion\;Height$$

$$Lesion\;volume\;ratio=\frac{Lesion\;volume}{Prostate\;volume}\;x\;100\%$$

Furthermore, seven risk calculators were evaluated. The Prostate Biopsy Collaborative Group (PBCG) model,^27^ the Mehralivand et al. model,^28^ the Mount Sinai Prebiopsy Risk Calculator (MSP-RC),^29^ the Prospective Loyola University multiparametric MRI (PLUM),^30^ the European Randomised Study of Screening for Prostate Cancer risk-calculator (ERSPC-RC),^31^ the van Leeuwen et al. model,^32^ and the Perez et al. model,^33^ were considered. Capio PCC dataset lacked the information on LV and LR biomarkers, and essential variables for the risk calculators including suspicious findings on digital rectal examination.

### Dataset generation and sensitivity analysis

The three datasets following alignment to the inclusion and exclusion criteria were merged into a combined dataset. A complete-case dataset was generated by excluding the observations with missing information in their PSA, Stockholm3 test, and prostate volume. The missingness was assumed to be completely at random. To mitigate selection bias and enhance internal validity, statistical analysis was first conducted in STHLM3-MRI RCT, then confirmed in the combined dataset. Moreover, multiple imputations with 50 imputations were performed, and the results were contrasted to the complete-case analysis. Imputation of continuous variables employed predictive mean matching, while binary variables employed logistic regression. In the primary analysis, only men with PSA ≥3 ng/mL were considered. In the secondary analysis, all men with PI-RADS 3 score were included. Additional statistical considerations and sample size justification are detailed in the Supplementary File.

### Statistical analysis

Participant characteristics were reported in terms of mean and standard deviation (SD) for continuous variables and counts with percentages for categorical variables.

To evaluate the discriminatory ability of the biomarkers to detect csPCa, the Area Under the Curve (AUC) of the Receiver Operating Characteristic curve (ROC) was computed using logistic regression. Individual biomarkers were first tested as continuous variables, then assessed based on literature-described cut-off points. Data-driven approaches included testing of binary cut-off points generated through Youden's index and Liu's index for each individual biomarker. The Youden's index (J) is defined as the sum of sensitivity and specificity minus one (J = Sensitivity + Specificity–1), while Liu's index is calculated as the product of sensitivity and specificity (Liu's index = Sensitivity x Specificity), both used to evaluate the performance of the diagnostic tests. Multiple logistic regressions were then employed to generate a predicted csPCa probability score, which was evaluated for its discriminatory ability using AUC. Bootstrapping was performed and the bias-corrected confidence interval was reported. Risk calculators were recalibrated to STLHM3-MRI study cohort. The combinations of biomarkers were tested both in the continuous format, and in binary format using the best performing cut-off points.

In addition to the predictive capacity, the corresponding sensitivity and specificity of detecting csPCa was reported with the proportion of biopsies performed, detected ISUP 3+, ISUP 1 and ISUP 0 cases. Two-sided 95% confidence intervals for proportions were calculated using the binomial exact method.

Decision curve analysis (DCA) graphs were computed both for net benefit and reductions in interventions for best-performing model and tested variables based. Operationalization of the best-performing model based on AUC, sensitivity, and specificity was performed in a clinical decision-making matrix. The reporting of the sensitivity, specificity, and the DCA was considered for STHLM3-MRI first-round. As for the combined dataset, based on the comparison between the complete-case and imputed datasets, the complete-case would be prioritized if the findings were similar between the two.

The reporting of the study confined by the transparent reporting of a multivariable prediction model for individual prognosis or diagnosis (TRIPOD) statement checklist. Additional data analysis considerations including model building strategy are available in the Supplementary File.

### Role of the funding source

The funders had no role in the design and conduct of the study; collection, management, analysis, and interpretation of the data; preparation, review, or approval of the manuscript; and decision to submit the manuscript for publication. The authors had full access to the data of the study, and all authors (AA, ME, AL, AD, LB, TP, JCE, FJ, UF, HG, TN) had the final responsibility for the decision to submit for publications.

## Results

A total of 900 participants with PSA ≥3 ng/mL and PI-RADS score of 3 were included in the study. Table 1 summarizes their demographic and clinical information. Total average age was 66 (SD 7.00), while mean PSA was 6.89 ng/mL (SD 5.91). Average prostate volume was 51 mL (SD 27.37), while PSAD was 0.16 (SD 0.17). Average MRI lesion volume in PI-RADS 3 was 0.36 mL (SD 0.75) in STHLM3-MRI first-round, and MRI lesion ratio was 0.85% (SD 1.37). Overall, 395 (4a%) had a benign result on their biopsy, 135 (15%) had ISUP1, while 370 (41%) were found to have csPCa, but only 75 (8%) had ISUP3+. Of the 900 men, 656 had complete information on their PSA, prostate volume, and Stockholm3 test (Fig. 1). Baseline characteristics of the 1146 men in the sensitivity analysis are shown in eTable S3.

**Table 1:** Demographic and clinical characteristics of men with PI-RADS 3 and had PSA ≥3 ng/mL.

	STHLM3-MRI First-round	STLHM3-MRI second-round	Capio PCC	Total
	n = 169 (19%)	n = 72 (8%)	n = 659 (73%)	n = 900 (100%)
Age in years, mean (SD)	65 (6.35)	66 (7.01)	66 (7.16)	66 (7.00)
PSA (ng/mL), median (IQR)	4.3 (3.5, 6.0)	3.9 (6.4, 5.2)	5.6 (4.1, 8.5)	5.1 (3.8, 7.6)
Not reported	0 (0%)	0 (0%)	42 (6%)	42 (5%)
Prostate volume (in mL), median (IQR)	41 (31, 57)	44.5 (35, 62)	46 (34, 62)	45 (33, 61)
Not reported	0 (0%)	0 (0%)	12 (2%)	12 (1%)
PSA density, median (IQR)	0.11 (0.08, 0.14)	0.09 (0.07, 0.12)	0.12 (0.09, 0.19)	0.12 (0.0.09, 0.17)
Not reported	0 (0%)	0 (0%)	54 (8%)	54 (6%)
Stockholm3 test score, median (IQR)	14 (10, 24)	15 (10, 23)	20 (14, 30)	18 (12, 28)
Not reported	0 (0%)	0 (0%)	215 (33%)	215 (24%)
Stockholm3 Density, median (IQR)	0.36 (0.18, 0.63)	0.34 (0.21, 0.54)	0.46 (0.28, 0.78)	0.41 (0.25, 0.70)
Not reported	0 (0%)	0 (0%)	216 (33%)	216 (24%)
MRI lesion volume (in mL), median (IQR)	0.18 (0.11, 0.34)	0.19 (0.09, 0.36)	N/A	N/A
Not reported	0 (0%)	0 (0%)	659 (100%)	659 (73%)
MRI lesion ratio, median (IQR)	0.47% (0.24%, 0.87%)	0.36% (0.21%, 0.93%)	N/A	N/A
Not reported	0 (0%)	0 (0%)	659 (100%)	659 (73%)
Suspicious digital rectal examination finding, n (%)				
No	107 (63%)	22 (31%)	N/A	N/A
Yes	59 (35%)	40 (51%)	N/A	N/A
Not reported	3 (2%)	10 (14%)	659 (100%)	672 (75%)
Lowest mean apparent diffusion coefficient (in mm^2^/s) median (IQR)	0.930 × 10^−3^ (0.819 × 10^−3^, 1.027 × 10^−3^)	0.933 × 10^−3^ (0.871 × 10^−3^, 0.981 × 10^−3^)	N/A	N/A
Not reported	8 (5%)	3 (4%)	659 (100%)	670 (74%)
Diffusion-weighted imaging				
3	112 (68%)	55 (77%)	N/A	N/A
4	49 (30%)	15 (21%)	N/A	N/A
5	3 (2%)	1 (1%)	N/A	N/A
Not reported	5 (3%)	1 (1%)	659 (100%)	665 (74%)
ISUP, n (%)				
Benign	77 (46%)	30 (42%)	288 (44%)	395 (44%)
ISUP 1 (GG 6)	30 (18%)	12 (17%)	93 (14%)	135 (15%)
ISUP 2	51 (32%)	19 (26%)	225 (34%)	295 (33%)
ISUP 3	5 (3%)	3 (4%)	30 (5%)	38 (4%)
ISUP 4	3 (2%)	1 (1%)	13 (2%)	17 (2%)
ISUP 5	3 (2%)	7 (10%)	10 (2%)	20 (2%)
Clinically significant prostate cancer (ISUP 2+), n (%)				
No	107 (63%)	42 (58%)	381 (58%)	530 (59%)
Yes	62 (37%)	30 (42%)	278 (42%)	370 (41%)
High-grade prostate cancer (ISUP 3+), n (%)				
No	158 (93%)	61 (85%)	606 (92%)	825 (92%)
Yes	11 (7%)	11 (15%)	53 (8%)	75 (8%)

GG, Gleason grade; PCC, prostate cancer center; PSA, prostate specific antigen; ISUP, International Society of Urological Pathology; ng, nanogram; mL, milliliter; IQR, interquartile range.

Being novel biomarkers, eFigures S1 and S2 shows the histogram distribution of Stockholm3 density and lesion volume ratio. Outcomes of imputation are shown in eTable S9.

### Role of individual biomarkers

#### PSA density

PSA density's predictive capacity of csPCa was AUC 0.58–0.66 in the different datasets (Table 2). It performed congruently across the individual cohorts, with similar findings in the complete-case and imputed combined datasets (Table 2). When utilizing commonly described cut-off points for PSA density, they reduced the number of biopsies performed greatly. It ranged in STHLM3-MRI first-round from 21% (15%, 28%) reduction for 0.075, 42% (34%, 50%) for 0.10, 76% (69%, 83%) for 0.15, to 90% (84%, 94%) in 0.20 (Table 3). However, they were accompanied with poor sensitivity; PSAD of 0.10 would miss approximately third of the csPCa with sensitivity 69% (56%, 80%), decreasing to 18% sensitivity (9%, 30%) to detect csPCa at 0.20 cut-off point based on its performance in STHLM3-MRI first-round. When considering ISUP ≥3, 9% (0%, 41%) are missed at 0.075, 27% (6%, 61%) at 0.10, 45% (17%, 77%) at 0.15, and 64% (31%, 89%) at 0.20.

**Table 2:** Predictive capacity to detect clinically significant prostate cancer^a^ among patients with PSA ≥3 ng/mL and PI-RADS 3 in the three cohorts.

Models	STHLM3-MRI RCT first-round screening	STHLM3-MRI RCT second-round screening	Capio PCC	Combined dataset (complete-case)	Combined dataset (imputed)
	AUC (95% CI)	AUC (95% CI)	AUC (95% CI)	AUC (95% CI)	AUC (95% CI)
**Individual continuous variables**					
Age	0.56 (0.48, 0.65)	0.59 (0.50, 0.73)	0.56 (0.51, 0.62)	0.57 (0.52, 0.61)	0.57 (0.54, 0.61)
Prostate volume	0.69 (0.60, 0.77)	0.63 (0.50, 0.76)	0.62 (0.56, 0.68)	0.64 (0.59, 0.68)	0.65 (0.61, 0.69)
PSA density (PSAD)	0.66 (0.56, 0.74)	0.61 (0.48, 0.73)	0.58 (0.47, 0.63)	0.60 (0.55, 0.64)	0.63 (0.59, 0.66)
Stockholm3	0.67 (0.58, 0.76)	0.63 (0.49, 0.76)	0.59 (0.53, 0.64)	0.62 (0.57, 0.66)	0.60 (0.56, 0.64)
Stockholm3D	0.72 (0.64, 0.80)	0.65 (0.50, 0.75)	0.63 (0.58, 0.69)	0.66 (0.62, 0.70)	0.66 (0.62, 0.70)
Lesion volume (LV)^b^	0.45 (0.34, 0.56)	0.50 (0.39, 0.59)	N/A	N/A	0.51 (0.46, 0.56)
Lesion volume ratio (LR)^b^	0.63 (0.38, 0.72)	0.54 (0.43, 0.66)	N/A	N/A	0.56 (0.47, 0.65)
Digital Rectal Examination (DRE)	0.56 (0.50, 0.64)	0.63 (0.52, 0.74)	N/A	N/A	0.52 (0.47, 0.56)

Models	STHLM3-MRI RCT first-round screening	Combined dataset (complete-case)	Combined dataset (imputed)
	AUC (95% CI)	AUC (95% CI)	AUC (95% CI)
**Model based on dichotomized variables**^c^			
Age, prostate volume, Stockholm3D, LR	0.82 (0.75, 0.87)	N/A	0.69^e^ (0.65, 0.72)
Age, prostate volume, PSAD, and Stockholm3D	0.80^d^ (0.73, 0.86)	0.70 (0.66, 0.74)	0.69 (0.66, 0.73)
**Risk calculators**			
the Prostate Biopsy Collaborative Group (PBCG) model	0.71 (0.62, 0.79)	N/A	0.66 (0.63, 0.70)
Mehralivand et al. model	0.73 (0.64, 0.81)	N/A	0.68 (0.64, 0.72)
Mount Sinai Prebiopsy Risk Calculator (MSP-RC)	0.63 (0.54, 0.72)	N/A	0.62 (0.58, 0.66)
Prospective Loyola University multiparametric MRI (PLUM)	0.69 (0.60, 0.76)	N/A	0.67 (0.63, 0.71)
European Randomised Study of Screening for Prostate Cancer risk-calculator (ERSPC-RC)	0.60 (0.51, 0.69)	N/A	0.56 (0.51, 0.61)
van Leeuwen et al. Model	0.72 (0.62, 0.79)	N/A	0.67 (0.63, 0.71)
Perez et al. Model	0.64 (0.54, 0.72)	N/A	0.60 (0.55, 0.64)

LR, MRI lesion volume ratio; LV, MRI lesion volume; PSA, prostate specific antigen; PSAD, prostate specific antigen density; PCa, prostate cancer; PCC, prostate cancer center; PI-RADS, Prostate Imaging-Reporting and Data System; Stockholm3D, Stockholm3 density.

Complete-case considered the men with complete information on their PSA, prostate volume, and Stockholm3 test. Imputed dataset included 50 imputations performed using predictive mean matching for continuous variables and logistic regression for binary variables.

a Clinically significant PCa is prostate cancer with ISUP grade ≥2.

b The lesion volume (in mL) was computed using the volume of the suspicious lesion that facilitated the PI-RADS score of 3. The lesion volume ratio is calculated by dividing the MRI lesion volume (in mL) by the prostate volume (in mL).

c Dichotomized variables were generated using Youden's Index and/or Liu's Index. The choice of the cut-off point considered the AUC and sensitivity/specificity performance.

d PSAD not significant in the model.

e LR not significant in the model.

**Table 3:** Predictive table based on the performance of selected biomarkers in men with PSA ≥3 ng/mL and PI-RADS 3 score on MRI.

Consideration^a^, n (%; 95%)	Biopsies performed	Detected csPCa^b^	Detected ISUP 3+	Detected GG6	Avoided unnecessary biopsies (GG6 and lower)
**Combined dataset (complete-case)**					
Biopsy all	656 (100%; 99%, 100%)	258 (100%; 99%, 100%)	50 (100%; 93%, 100%)	105 (100%; 97%, 100%)	0 (0%; 0%, 1%)
PSAD ≥0.075	537 (82%; 79%, 85%)	223 (86%; 82%, 90%)	39 (78%; 64%, 88%)	92 (88%; 80%, 93%)	84 (21%; 17%, 25%)
PSAD ≥0.10	393 (60%; 56%, 64%)	174 (67%; 61%, 73%)	30 (60%; 45%, 74%)	65 (62%; 52%, 71%)	179 (45%; 40%, 50%)
PSAD ≥0.15	189 (29%; 25%, 32%)	96 (37%; 31%, 43%)	20 (40%; 26%, 55%)	29 (29%; 19%, 37%)	305 (77%; 72%, 81%)
PSAD ≥0.20	94 (14%; 12%, 17%)	56 (22%; 17%, 27%)	11 (22%; 12%, 36%)	13 (12%; 7%, 20%)	360 (90%; 87%, 93%)
Stockholm3 ≥11%	562 (86%; 83%, 88%)	237 (92%; 88%, 95%)	45 (90%; 78%, 97%)	90 (86%; 78%, 92%)	73 (18%; 15%, 23%)
Stockholm3 ≥13%	484 (74%; 70%, 77%)	213 (83%; 77%, 87%)	43 (86%; 73%, 94%)	79 (75%; 66%, 83%)	127 (32%; 27%, 37%)
Stockholm3 ≥15%	434 (66%; 62%, 70%)	203 (79%; 73%, 84%)	43 (86%; 73%, 94%)	75 (71%; 62%, 80%)	167 (42%; 37%, 47%)
Best performing model^c^	485 (74%; 70%, 77%)	231 (90%; 85%, 93%)	47 (94%; 83%, 99%)	79 (75%; 66%, 83%)	144 (36%; 31%, 41%)
Biopsy none	0 (0%; 0%, 1%^e^)	0 (0%; 0%, 1%^e^)	0 (0%; 0%, 7%^e^)	0 (0%; 0%, 3%^e^)	398 (100%; 99%^e^, 100%)

Consideration^a^, n (%)	Biopsies performed	Detected csPCa^b^	Detected ISUP 3+	Detected GG6	Avoided unnecessary biopsies (GG6 and lower)
**STHLM3-MRI RCT First-round**					
Biopsy all	169 (100%; 98%^e^, 100%)	62 (100%; 94%, 100%)	11 (100%; 72%, 100%)	30 (100%; 88%, 100%)	0 (0%; 0%, 3%)
PSAD ≥0.075	133 (79%; 72%, 85%)	55 (89%; 78%, 95%)	10 (91%; 59%, 100%)	27 (90%; 73%, 98%)	29 (27%; 19%, 37%)
PSAD ≥0.10	98 (58%; 50%, 66%)	43 (69%; 56%, 80%)	8 (73%; 39%, 94%)	22 (73%; 54%, 88%)	52 (49%; 39%, 58%)
PSAD ≥0.15	40 (24%; 17%, 31%)	23 (37%; 25%, 50%)	6 (55%; 23%, 83%)	6 (20%; 8%, 39%)	90 (84%; 76%, 90%)
PSAD ≥0.20	17 (10%; 6%, 16%)	11 (18%; 9%, 30%)	4 (36%; 11%, 69%)	3 (10%; 2%, 27%)	101 (94%; 88%, 98%)
Stockholm3 ≥11%	119 (70%; 63%, 77%)	51 (82%, 70%, 91%)	7 (64%; 31%, 89%)	20 (67%; 47%, 83%)	39 (36%; 27%, 46%)
Stockholm3 ≥13%	96 (57%; 49%, 64%)	45 (73%, 60%, 83%)	7 (64%; 31%, 89%)	15 (50%; 31%, 69%)	56 (52%; 42%, 62%)
Stockholm3 ≥15%	83 (49%; 41%, 57%)	43 (69%; 56%, 80%)	7 (64%; 31%, 89%)	13 (43%; 25%, 63%)	67 (63%; 53%, 72%)
Best performing model^d^	114 (67%; 60%, 74%)	61 (98%; 91%, 100%)	10 (91%; 59%, 100%)	19 (63%; 44%, 80%)	54 (50%; 41%, 60%)
Biopsy none	0 (0%; 0%, 2%^e^)	0 (0%; 0%, 6%^e^)	0 (0%; 0%, 28%^e^)	0 (0%; 0%, 12%^e^)	107 (100%; 97%^e^, 100%)

GG, Gleason grade; LR, MRI lesion ratio; LV, MRI lesion volume; PSA, prostate specific antigen; PSAD, prostate specific antigen density; PCa, prostate cancer; PI-RADS, Prostate Imaging-Reporting and Data System; Stockholm3D, Stockholm3 density.

a models compare to screening all patients with biopsies. Each column is compared separately.

b Significant PCa is prostate cancer with ISUP grade ≥2.

c Best performing model consist of prostate volume, age, Stockholm3D, and PSA density.

d Best performing model consist of prostate volume, age, Stockholm3D, MRI lesion volume ratio.

e Based on statistical computation, and might not be possible estimates in real-world setting.

The performance was similar in the combined dataset, with 18% (15%, 21%) reduction of 0.075, 40% (36%, 44%) for 0.10, 71% (68%, 75%) for 0.15, and 0.86% (83%, 88%) in 0.20. It showed 67% sensitivity (61%, 73%) at PSAD of 0.10, decreasing to 22% (17%, 27%) at PSAD 0.20. PSAD of 0.15 would miss 63% (57%, 69%) of csPCa, while avoiding 71% (68%, 75%) of unnecessary biopsies. Regarding the detection of ISUP ≥3, PSAD would miss 22% (12%, 36%) of high-grade PCa at PSAD 0.075, 40% (26%, 55%) at 0.10, 60% (45%, 74%) at 0.15, and 78% (64%, 88%) at 0.20 based on the combined dataset.

**Fig. 2:**
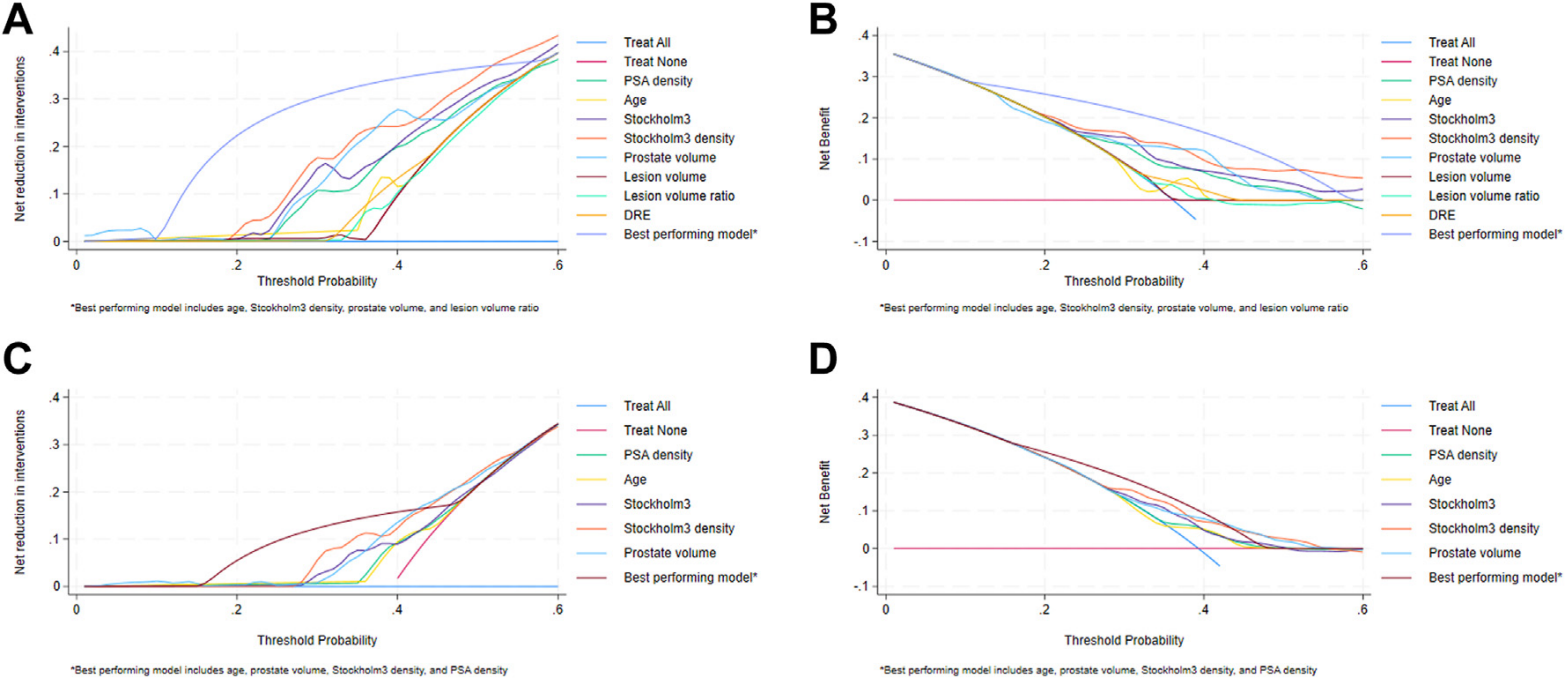
Decision curve analysis for clinically significant prostate cancer among men with PI-RADS 3 and had PSA of 3 ng/mL or higher. Description: Decision curve analysis of individual biomarkers and best performing models in combined dataset (complete-case) and STHLM3-MRI first-round for men with PSA ≥3 ng/mL and PI-RADS 3 lesion on MRI. A. Shows net reductions in the combined dataset (complete-case), B. Shows net reductions in STHLM3-MRI first-round, C. Shows net benefit in the combined dataset (complete-case), D. Shows net benefit in STHLM3-MRI first-round. Colours in A. and B. blue: treat all, red: treat none, green: PSA density, yellow: age, purple: Stockholm3 test, orange: Stockholm3 density, light blue: prostate volume, maroon: lesion volume, cyan: lesion volume ratio, light orange: suspicious digital rectal examination, violet: best performing model (age, Stockholm3 density, prostate volume, lesion volume ratio). Colours in C. and D. blue: treat all, red: treat none, green: PSA density, yellow: age, purple: Stockholm3 test, orange: Stockholm3 density, light blue: prostate volume, maroon: best performing model (age, Stockholm3 density, prostate volume, PSA density).

Upon comparing the performance of PSAD in all men with PI-RADS 3 (sensitivity analysis), PSAD had lower predictive capacity and sensitivity, but slightly higher specificity (eTables S4 and S8). DCA graphs show a modest net benefit of 0.2 at threshold 0.4 from using PSA density alone (Fig. 2), a similar finding was observed in the sensitivity analysis dataset (eFigure S3).

#### Role of other biomarkers

The AUC of Stockholm3 ranged between 0.58 and 0.66 among the datasets (Table 2). Stockholm3 score of 11% would miss 18% (9%, 30%) of the csPCa cases and avoid 30% (23%, 37%) of the unnecessary biopsies based on STHLM3-MRI first-round (Table 3). Stockholm3 score of 13% performed similarly as PSAD of 0.10 but with higher sensitivity (73%; 60%, 83%). Stockholm3 density had the highest AUC for a univariate model with AUC of 0.72 (0.64, 0.80) in STHLM3-MRI first-round (Table 2). MRI lesion volume had poor AUC 0.45 (0.36, 0.54), with little benefit to multivariable models (eTables S4 and S5). In contrast, MRI lesion volume ratio performed better with large confidence interval and an AUC of 0.63 (95% CI 0.38, 0.72), and the data-driven cut-off point of 0.6% was used for testing in the multivariable models (eTables S1 and S6). Men with smaller prostates had higher risk for csPCa. Suspicious findings on digital rectal examination (DRE) had modest predictive capacity of 0.56 (0.50, 0.64) in STHLM3-MRI first-round (Table 2). Upon evaluating net benefit in DCA graphs, prostate volume and Stockholm3 density showed the highest net benefit of any individual biomarker (Fig. 2).

#### Multivariable models

The predicative capacity of the seven tested risk calculators in STHLM3-MRI first-round ranged from 0.60 to 0.73, with Mehralivand et al. model performing the highest (Table 2). Probability models using continuous variables did not show substantial improvement over univariate models (eTable S4). In STHLM3-MRI first-round, the best performing model had a higher AUC of 0.82 (0.75, 0.87), and included age, prostate volume, Stockholm3D, and LR. The performance of the models in terms of sensitivity and specificity was similar across STHLM3-MRI and combined dataset when comparing models with variables available in the combined dataset (eTables S4 and S5).

The best-performing multivariable model in the combined dataset (complete-case) had an AUC of 0.70 (0.66, 0.74) and included age, prostate volume, PSAD and Stockholm3D, which the imputed dataset showed similar findings (AUC 0.69; 0.66, 0.73) (Table 2).

The best-performing models were further optimized based on the sensitivity and specificity of each cut-off point (eTables S2 and S7). In STHLM3-MRI first-round when evaluating the clinical effects, the best performing model has 98% sensitivity and 50% specificity, reducing the total biopsies by 33% at the optimal cut-off point (Table 3 and eTable S2). When contrasting these findings to the sensitivity analysis (entire dataset of men with PI-RADS 3 screened using Stockholm3 as a reflex test if PSA ≥1.5 ng/mL), performance of the biomarkers was comparatively lower, and the best-performing model benefited from adding DRE to the best-performing model to yield similar AUC of 0.77 (95% CI 0.71, 0.83), with 90% sensitivity (83%, 96%) and 47% specificity (38%, 54%) (eTables S4, S5, S7 and S8).

DCA graphs show larger benefit from the best-performing models compared to individual biomarkers, with higher net benefit and avoided biopsies in STHLM3-MRI first-round (Fig. 2). Synthesizing the information from the main and sensitivity analysis, the models were operationalized in a decision-making workflow illustrated in eFigure S4.

## Discussion

In men with a PI-RADS score of 3, about one-third were found to have ISUP ≥2 (39%), but only 8% had ISUP ≥3 prostate cancer. When evaluating the predictive capacity of regularly described biomarkers in this dataset including screening-by-invitation, repeat screening and a clinical cohort, none of the individual biomarkers alone achieved satisfactory performance to identify men with significant prostate cancer. However, when considering complex multivariate models of several biomarkers, the predictive capacity performed satisfactorily with potential after validation for clinical application when optimized for the cut-off point.

Currently described thresholds for PSA density showed suboptimal performance among men with a PI-RADS 3 lesion by missing many clinically significant prostate cancers. Previous publications suggest to only consider biopsy if PSAD ≥0.10.^12^^,^^13^ These recommendations were adopted by the EAU and Swedish guidelines,^9^^,^^10^ who recommend considering biopsy in patients with PI-RADS 3 only if the they have PSAD ≥0.10, and perform it at threshold ≥0.20. In our data, PSAD ≥0.10 would miss at least a third of the csPCa cases as demonstrated by the main and sensitivity analysis. When considering high-grade PCa, the limitations of PSAD persisted, with similar ratios of ISUP ≥3 would be missed as the ISUP ≥2 (Table 3 and eTable 8). Sigle et al., specifically studied different biomarkers in men with PI-RADS 3 score.^13^ They described an AUC of PSA density of 0.67,^13^ which is slightly higher than the findings in our study (0.66 in STHLM3-MRI first-round and 0.60–0.63 in the combined dataset, Table 2). However, in their study they selected patients diagnosed from 2012 to 2021, subject to different PI-RADS criteria versions, without external validation of the readings.^13^ Moreover, many of the studies that described or developed the tools for detecting csPCa using PSAD or risk calculators commonly relied solely on center-based data^12^^,^^14^ could suffer from selection bias due to the nature of the study design. Another interesting finding was that the performance of PSAD was worse in detecting csPCa among men with non-elevated PSA but elevated Stockholm3 test (eTable S8).

Even when considering a more holistic tool like Stockholm3 that combines genetic information, protein biomarkers, and clinical variables^34^ did not reach on its own optimal performance in terms of sensitivity and specificity in men with PI-RADS 3, even though it outperformed PSA density in detecting csPCa. While genetic markers can assist in the diagnosis of csPCa,^34^ their predictive accuracy vary from one model to another. For example, the urine-based genetic test SelectMDx showed inferior performance in comparison to mp-MRI in detection of csPCa.^35^ In contrast, Stockholm3 test and systematic biopsy performed similarly to MRI followed by combined targeted and systematic biopsy in detection of csPCa.^36^

Novel tools like Stockholm3 density and MRI lesion volume ratio, while insufficient alone, improved predictive capacity when combined with clinical variables like prostate volume. The best model using dichotomized variables achieved an AUC of 0.82 (0.75, 0.87) could reduce biopsies by a third while virtually capturing all csPCa cases among men with PSA ≥3 ng/mL. The best-performing multivariable models might have potential for clinical application, by reducing the number of biopsies performed and detection of unnecessary biopsies, while maintaining high sensitivity for csPCA. However, the inherit complexity of operationalization and the need to perform several tests can limit its usage, further requiring external validation and assessment of cost-effectiveness. Risk calculators had lower AUC compared to the best-performing model, and performed similarly to Stockholm3 density on its own.

In the face of multiplex tools, while having individual biomarkers insufficiently detecting csPCa and missing high-grade PCa (ISUP ≥3), biopsying all men with PI-RADS 3 might be an alternative to avoid missing large numbers of csPCa.

In our study, 39% of the men with PI-RADS 3 score had csPCa, which is on the higher limit of the reported studies' average.^6^, ^7^, ^8^ External review of random MRI readings was performed, and the MRI readings were conducted in the three cohorts by the same radiologist who has long experience reading prostate MRIs, following a modified PI-RADS protocol v2.1 using bp-MRI.^22^ The readings had high concordance of 83% (82 of 99) cases reviewed by the external uro-radiologist, supporting high-confidence in the used PI-RADS scoring.^22^ The usage of bp-MRI was found to be non-inferior to mp-MRI in the diagnosis of prostate cancer in multi-centre European study.^37^ However, the usage of bp-MRI might include some PI-RADS 4 as PI-RADS 3 due to the omission of contrast.^38^ External validation by expert radiologist was not performed by other studies.^6^^,^^12^, ^13^, ^14^^,^^30^ Radiologists’ training and clinical experience could influence their decision to mark the score as PI-RADS 3 or less,^16^^,^^17^ which might inflate the number of non-csPCa due to cautious scoring. Due to the inter-observer variability, reproducibility and transferability of diagnostic models can be limited to other clinical and screening settings, as the models depend on the knowledge, training, and experience of the performing radiologist.^16^^,^^17^^,^^22^ Moreover, the utilization of MRI parameters and outputs such as the apparent diffusion coefficient (ADC) might assist in the differentiation between benign and cancerous lesions.^39^ Moreover, the use of PSMA PET/CT as a possible alternative to MRI for evaluating PI-RADS scores may help improve accuracy of detecting csPCa.^40^ Artificial intelligence (AI) usage can provide an opportunity to overcome this limitation through AI-assisted radiology (i.e., AI + human radiologist).^41^^,^^42^

Our study indicates that currently suggested tools in the clinical guidelines,^9^^,^^10^ research studies,^12^^,^^14^^,^^30^ and organized testing^43^ would insufficiently perform in terms of detecting csPCa and high-grade prostate cancer among men with PI-RADS 3. The performance of PSAD and the other individual biomarkers was consistently found to be suboptimal in all assessed contexts; screening-by-invitation, re-screening and in clinical-practice cohorts both in men with PSA ≥3 ng/mL and in men with PSA ≥1.5 ng/mL and Stockholm3 test ≥11%. This warrants careful re-examination of their “in silo” usage in clinical practice. However, our proposed model based on STHLM3-MRI RCT, leverages novel biomarkers, might assist in bridging the current clinical gap by reducing biopsies performed without sacrificing the sensitivity to detect csPCa. When faced with limitations of usage and benefits of biomarkers in detecting csPCa, our findings highlight the importance of high-quality radiological evaluation in order to limit the number of men with lesions assessed as equivocal. This could be achieved with quality training and experience of radiologists, and potential usage of AI-assisted radiology.^16^^,^^17^^,^^41^^,^^42^

The use of three cohort datasets enabled reflection of common clinical scenarios from screening-by-invitation, rescreening, and clinical-practice. With 1146 men with a PI-RADS score of 3 (of which 900 men with PSA ≥3 ng/mL), this study includes the largest non-meta-analysed individual-level single-screening protocol dataset in any published study on this topic.^6^^,^^12^, ^13^, ^14^^,^^30^ The usage of a wide scope of biomarkers covering genetic polymorphisms, protein and blood biomarkers, and clinical variables ensured considerations of several potential tools frequently used and described. Furthermore, the MRI readings were externally validated with quality assurance of their scoring.^22^ Usage of centre-based data or rescreening alone might affect the internal validity of the study due to selection-by-indication and the characteristics of the participants.^18^^,^^44^ To address potential selection bias, we compared results from the combined dataset to the STHLM3-MRI clinical trial. Another limitation was incomplete biomarkers data collected from Capio PCC, which limited the testing of risk calculators, lesion ratio, lesion volume, and DRE in the combined dataset without reliance on multiple imputations. Unmeasured and/or unavailable biomarkers not included in the study may affect the inferences made due to omitted predictors bias. Moreover, the chosen statistical model may not fully capture the complex relationships between the predictor variables and the outcome. In our study, we reported the sensitivity and the specificity of the models reflecting the tests’ performance, while in practice, the positive predictive value (PPV) and the negative predictive value (NPV) are more relevant reflecting diagnosis outcomes.^45^ However, they are influenced by the true prevalence in the population.^45^ Considering that the quality of MRI readings varies significantly between radiologists,^16^^,^^17^^,^^22^ and the reported prevalence of csPCa among men with PI-RADS score 3,^6^, ^7^, ^8^ the reporting of the sensitivity and specificity might be more favourable.^45^ The high observed number of csPCa detected in men with PI-RADS 3 might be a consequence of using an ADC-value cut-off for including a suspicious lesion for biopsy and not related to competence of the readers or image quality.^44^ The ADC cut-off might explain the relatively high outcome of csPCa. Moreover, the usage of bp-MRI instead of mp-MRI might misclassify some of the PI-RADS 4 cases as PI-RADS 3 cases due to the absence of the dye.^44^ However, our study still falls within the described range from other reported studies that reported the detection of csPCa among PI-RADS 3.^6^, ^7^, ^8^ Although current practice refers patients for MRI following suspicious findings on DRE and PSA dynamics,^10^ the men in our study proceeded to MRI following raised PSA. With the European Commission updating in December 2022 the European Union council screening recommendations on organized cancer screening to include prostate cancer screening,^46^ the usage of PSA ≥3 ng/mL is likely to be the main method of screening.^43^ This makes our study design ideal as it provides continued guidance and relevance for future studies. In addition, the STHLM3-MRI clinical trial is planning to amend its protocol to consider performing the prostate imaging quality scoring system second version (PI-QUAL v2) in the MRI quality assurance step. Considering that this study is a validation study for predefined biomarkers, and an exploratory study for novel biomarkers and multiplex models, validation of the emerging findings would be required.

In conclusion, the presence of high-grade prostate cancer is uncommon in men with PI-RADS score of 3. Literature-described biomarkers and their thresholds (namely PSAD) are insufficient alone in detecting csPCa or high-grade prostate cancer in men with PI-RADS score of 3. Usage of several biomarkers might assist in clinical decision to biopsy. Future studies should validate these findings. Caution should be exerted when using PSAD alone to ascertain biopsy decisions and deferral of screening.

## Contributors

TN conceptualized and devised the study, AA, ME, HG, TN designed the study, AA conducted data analysis, AA, AD, TN accessed and verified the data, AA, ME, AL, AD, LB, TP, JCE, FJ, UF, HG, TN interpreted the data, AA, TN drafted the manuscript, AA, ME, AL, AD, LB, TP, JCE, FJ, UF, HG, TN critically reviewed the manuscript, and gave final approval and agreement to accountability of all aspects related to the manuscript, TN supervised the work of the study. All authors read and approved the final manuscript for submission.

## Data sharing statement

The datasets generated and/or analysed in the current study are not publicly available due to ethical and data sharing restrictions/laws, including but not limited to GDPR. The data, however, can be requested formally from the principal investigators of each dataset. The formal request entails proposal submission, approval of the proposal, acquiring Swedish ethics committee approval, and signed research collaboration agreement and data sharing agreement. Following attainment of the aforementioned steps, the data can be shared.

## Declaration of interests

TN reported grants from the Swedish Research Council, Swedish Cancer Society, and Region Stockholm Research Funding (ALF), personal fees from Ipsen and AstraZeneca, and owning stock in A3P Biomedical AB outside the submitted work. HG reported owning stock, being a board member, and holding patents in A3P Biomedical AB outside the submitted work. ME reported grants from the Swedish Research Council, Swedish Cancer Society, and Swedish Prostate Cancer Society during the conduct of the study as well as holding patents in A3P Biomedical AB outside the submitted work, being a stockholder of A3P Biomedical AB and Clinsight AB, and has received speaker fees for scientific events from Ipsen and Johnson & Johnson. HG and ME hold the following patents (Method for indicating a presence or non-presence of aggressive prostate cancer WO2013EP74259; 20131120, Prognostic method for individuals with prostate cancer WO2013EP74270; 20131120, Method for indicating the presence or non-presence of prostate cancer WO2013SE50554; 20130516), while HG solely holds the following patents (Method for indicating a presence of prostate cancer in individuals with particular characteristics WO2018EP52473; 20180201, Method for detecting a solid tumour cancer WO2015SE50272; 20150311). FJ has received speaker fees for a scientific event from AstraZeneca. AL reported grants from the Swedish Cancer Society, the Swedish Prostate Cancer Association, Magnus Bergvall Foundation, Ahréns foundation, and has received speaker fees for scientific events from Janssen, Ipsen, Johnson & Johnson, Bayer, and Accord Health Care AB. TP is employed by A3P Biomedical AB.

AA, AD, LB, JCE, and UF declare no competing interests. No other conflicts of interest were reported.

## Appendix A Supplementary data

Supplementary Document

Summary in Swedish
