# Supplementary material for: Evaluating the performance of existing tools to predict clinically significant prostate cancer in men with indeterminate lesions on biparametric MRI and development of a novel multiplex model: a prospective cohort study
Source: eClinicalMedicine. 2025 Apr 3;82:103191. doi: 10.1016/j.eclinm.2025.103191 (PMC12002877; doi:10.1016/j.eclinm.2025.103191)

**Evaluating the performance of existing tools to predict clinically significant prostate cancer in men with indeterminate lesions on biparametric MRI and development of a novel multiplex model: a prospective cohort study**

**Supplementary File**

Ahmad Abbadi^1^ MD, Martin Eklund^1^ PhD, Anna Lantz^1,2^ MD, Andrea Discacciati^1^ PhD, Lars Björnebo^1^ MD, Thorgerdur Palsdottir^1^ PhD, Jan Chandra Engel^1,3^ MD, Fredrik Jäderling^2,4^ MD, Ugo Falagario^1,5^ MD, Henrik Grönberg^1,6^ MD, Tobias Nordström^1,3^ MD

**Affiliations**

1. Department of Medical Epidemiology and Biostatistics, Karolinska Institutet, Solna, Sweden
2. Department of Molecular Medicine and Surgery (Solna), Karolinska Institutet, Stockholm, Sweden
3. Department of Clinical Sciences at Danderyds Hospital, Karolinska Institutet, Solna, Sweden
4. Department of Radiology, Capio S:t Görans Hospital, Stockholm, Sweden
5. Department of Urology and Kidney Transplantation, University of Foggia, Foggia, Italy
6. Department of Oncology, Capio S:t Görans Hospital, Stockholm, Sweden

**Correspondence to**

Ahmad Abbadi, MD MMedSc

Department of Medical Epidemiology and Biostatistics, Karolinska Institutet, Solna, Sweden

Nobels väg 12A, 171 65 Solna, Sweden

[ahmad.abbadi@ki.se](mailto:ahmad.abbadi@ki.se)

**Methods**

STHLM3-MRI clinical trial first- and second-rounds:

The STHLM3-MRI clinical trial is a prospective, population-based, open-label, and non-inferiority trial. ^1^ It screened men aged 50-74 living in Stockholm using PSA and Stockholm 3 tests, followed by randomization to either systematic biopsies (standard arm) or targeted and systematic biopsies for MRI-positive men with PI-RADS score ≥ 3 (experimental arm). Men were considered positive for screening if their PSA was ≥ 3 ng/mL, or if they had PSA ≥ 1.5 ng/mL and a Stockholm 3 test risk ≥ 11%. Positive screening led to a biparametric MRI.

Men with PSA ≥ 1.5 ng/mL and no cancer detected at the first screening round were invited for second-round screening about 2 years after initial enrolment ^2^. Screening in the second round followed the same procedures and interventions as in the first round.

First-round screening was conducted between 5^th^ of February, 2018, and 4^th^ of March, 2020, while second-round screening was conducted between 10^th^ of November, 2021, and 20^th^ of February, 2023.

The data from STHLM3-MRI was retrieved directly from the participating laboratories, medical charts, and filled out forms by the participants.

Capio S:t Görans Prostate Cancer Centre cohort:

Men who self-initiated prostate cancer screening or were referred to Capio S:t Görans Prostate Cancer Centre underwent PSA testing and/or Stockholm 3 test. Similar to STHLM3-MRI clinical trial, men with PSA ≥ 3 ng/mL, or had PSA ≥ 1.5 ng/mL and a Stockholm 3 test risk ≥ 11%, were further screened by biparametric MRI. MRI-positive men with PI-RADS score ≥ 3 proceeded to systematic and/or targeted biopsy. Men screened by PSA and/or Stockholm 3 were included from 5^th^ of January 2017 to end of June 2023. This referral hospital followed closely the MRI and biopsy protocols as the STHLM3-MRI clinical trial. The data was retrieved directly from the laboratories and medical charts.

Eligibility to STHLM3-MRI clinical trial:

Men aged 50-74 living in Stockholm in 2018 were randomly invited to participate through mail invitations by Statistics Sweden (SCB). ^1^ Men who previously underwent prostate biopsy within 60 days of the invitation, had a confirmed diagnosis of prostate cancer, had contradictions for MRI, or had severe chronic illnesses (i.e., metastatic cancer, dementia, or severe cardiovascular disease) were excluded from the study. Eligible men underwent PSA and Stockholm 3 tests, with randomization based on blood test results in a 2:3 ratio (standard arm : experimental arm). For the second-round screening, all men from the first round with PSA ≥ 1.5 ng/mL were reinvited ^2^, adhering to the initial exclusion criteria. Only men in the experimental arm with a PI-RADS score of 3 were included in this study.

Clinical procedure of STHLM3-MRI clinical trial and Capio S:t Göran’s Hospital:

*Blood testing:*

Participants had blood drawn into 12 mL Ethylenediaminetetraacetic acid (EDTA) tube within one of the 60 laboratories in Stockholm, and samples were sent for analysis of PSA and Stockholm 3 test at the A23 laboratory (A3P Biomedical, Uppsala, Sweden) using reagents from Thermo Fisher Scientific (Waltham, Massachusetts, USA) ^1,2^. Both in the first- and second-round screening, PSA was analysed in the same laboratory using the same technique (Kryptor Compact Plus from Thermo Fisher Scientific).

Men with positive tests proceeded to perform MRI and/or biopsy as per randomization assignment into one of the three centres specified in the study protocol. ^3^ All men in Capio S:t Göran’s Hospital were offered MRI screening before proceeding to biopsy.

*MRI procedure:*

Men in the experimental arm and in Capio S:t Göran’s Hospital proceeded to do an MRI using either 1.5-Tesla (T) Magnetom (Siemens Healthcare, Germany) or a 3T Signa scanner (GE Healthcare, USA). Using a biparamatric short protocol, diffusion-weighted and T2-weighted images were acquired. The lesion suggestive of prostate cancer was scored using modified PI-RADS version 2.1. ^1^ A random sample of MRIs were externally validated by Dr. Jonas Wallström (Gothenburg University Hospital), while the MRIs in the study were read by the same radiologist who examined the MRIs in Capio S:t Göran’s Hospital. ^4^ Of the 99 randomly selected samples, 82 (83%) were aligned, showing high concordance, with Cohen’s kappa statistic of 78% for PI-RADS v2.1. ^4^ For the STHLM3-MRI second-round, approximately 10% of the negative cases (PI-RADS 1-2) and all positive cases PI-RADS ≥3 were double-read. ^2^ The two readers are radiologists with more than 7 years of experience in prostate MRI reading at the time of first-round study. For cases in the Capio PCC, all cases were double-read by at least one radiologist with more than 7 years’ experience in prostate MRI reading. The outcomes of the radiology quality control are available in **Appendix 1**.

*Biopsy procedure:*

Men with PI-RADS scores of ≥ 3 proceeded to have targeted and systematic biopsies in a single session. Fusion targeted biopsies were performed with 4 cores per suspicious lesion, while systematic biopsies were performed with 10-12 cores. Systematic biopsies were performed for men in the standard arm using the Swedish national template of 10-12 biopsy cores following the national guidelines. ^5^

*Pathology procedure:*

Biopsies were histopathologically assessed at the Unilabs uropathology unit in Stockholm. ^1^ For each biopsy, Gleason score with corresponding International Society of Urological Pathology (ISUP) grade, percentage of Gleason grade 4 in cancerous lesion, and size (in mm) of cancerous lesion were reported according to ISUP guidelines. ^6^

Full description of the MRI, biopsy, and pathology procedures are detailed elsewhere for STHLM3-MRI clinical trial, ^1,2,4^ and match closely the procedures performed at Capio S:t Göran’s Hospital.

Sample size calculation:

The suggested sample size considered the events per variable (EPV) method, first computed with 10 EPV, and then relaxing the assumption 20 and 50 EPV. ^7^ We considered 8 predictors for computation (age, prostate volume, Stockholm3, Stockholm3 density, PSA density, MRI lesion volume, MRI lesion volume ratio, and digital rectal examination), but a maximum of 6 would be considered in the same model (either Stockholm3 or Stockholm3 density, and either MRI lesion volume and MRI lesion volume ratio). The proportion of events was conservatively estimated at 0.3, considering the published numbers from STHLM3-MRI trial results (0.37). ^1^ The sample size based on 10 EPV and 6 predictors was 200, while 20 EPV was 400, and 50 EPV was 1,000. The STHLM3-MRI first-round has sufficient number of participants to provide sufficient power based on 10 EPV, but for higher number, the combined dataset is required to achieve the recommended number.

Additional data analysis considerations:

In the complete-case analysis, considerations of risk calculators, LV, LR, and DRE were only performed using the first-round of STHLM3-MRI clinical trial. Without Capio S:t Göran’s Hospital dataset, the second-round data was not included in the testing of these additional variables due to selection bias considerations; as it contains a special population of men who were previously screened and tested positive after 2 years from initial assessment. ^2^ In contrast, the imputed dataset utilized the information available and performed 50 imputations on the combined dataset. The imputation declared the age, having csPCa, the ISUP score, and the site of the study as variables with complete information, while the Stockholm3 test, PSA, prostate volume, and lesion volume were chain imputed using predictive mean matching, and digital rectal examination findings, family history, and previous biopsy status using logistic regression. These variables were also required for the risk calculators’ computation. Rubin’s rules were used to adjust for the variance in the imputed dataset analysis.

Assessment of the linearity assumption for the continuous predictors (age, stockholm3 test, prostate volume, and lesion volume) in the logistic regression was performed using spline transformations and likelihood ratio tests. For each predictor, we compared a model with restricted cubic splines to a model with the original linear term. The number of knots tested were 3, 4, 5, and 6. The knot locations were based on the recommended percentiles described by Harrell et al. ^8^ The linear model was deemed better than the model with restricted cubic splines if the likelihood ratio test was > 0.05, and the Akaike information criterion (AIC) and the Bayesian information criterion (BIC) were lower.

Non-parametric bootstrapping with 1,000 iterations was applied to the logistic regression model, including probability prediction and the computation of the area under the curve (AUC). We report the bias-corrected AUC obtained from the bootstrapping process.

The bootstrapping approach was incorporated in the handling of continuous variables, risk calculators, and multivariable models. However, when model selection decisions were influenced by both clinical reasoning and data-driven considerations, it was not feasible to replicate the exact selection process within each bootstrap iteration. As a result, we performed bootstrapping on the final models chosen based on complete-case and imputed datasets rather than reapplying the model selection process in each resampling iteration.

*Model building strategy:*

Model development was conducted using a combination of theory-driven and data-driven approaches. Initially, clinically relevant biomarkers identified through a comprehensive literature review, ^1,9-13^ along with novel biomarkers, were assessed for their individual predictive accuracy using univariable logistic regression.

To develop multivariable prediction models, a two-stage approach was employed. First, a base model was constructed using variables selected a priori based on clinical knowledge and established associations with the outcome of interest. This approach prioritizes biological plausibility and avoids purely data-driven model building, which can lead to overfitting and reduced generalizability. ^14^

Subsequently, a backward elimination procedure was used to refine the base model. This procedure started with all variables in the base model and iteratively removed the variable with the highest p-value, provided that its removal did not significantly decrease the model's predictive performance as assessed by the change in the area under the receiver operating characteristic curve (AUC). A p-value of 0.2 was used in the backward elimination procedure initially. This approach balances clinical relevance with statistical parsimony. It is crucial to note that p-values were used primarily as a guide for variable removal, not as a strict criterion for inclusion or exclusion. The primary aim was to identify a model with optimal predictive performance using a minimum set of clinically meaningful predictors, mitigating concerns about testimation bias associated with sole reliance on p-values. ^8^

Continuous predictors were initially evaluated in their continuous form. Where clinically justified, literature-based cut-off points were then tested. Additionally, data-driven cut-off points were determined using Youden’s index and Liu’s index, and these were also evaluated. Youden’s index maximizes the sum of sensitivity and specificity, providing a cut-off that balances both measures. ^15^ Liu’s index, on the other hand, is useful when dealing with skewed data. ^16^ Both indices offer data-driven approaches to determining optimal cut-off points, complementing clinically relevant and literature-based thresholds. All models were assessed for their predictive accuracy using the AUC.

These steps were carried in the STHLM3-MRI first-round and the combined dataset, both in the complete-case analysis and imputed combined dataset. Furthermore, to ensure that the datasets were similar in terms of predictive capacity, the individual biomarkers were tested for their predictive capacity in the three individual datasets.

Due to power considerations, the main analysis was limited to men with PSA ≥3ng/mL with no further stratifications. Similarly, the secondary analysis considered the entire dataset (all men with PI-RADS 3 score), and did not perform stratified analysis (i.e., PSA ≥3ng/mL and <3ng/mL with Stockholm3 test ≥11%) as there are few men in the latter category, limiting the potential inferences made based on that analysis.

When selecting best-performing models, sensitivity threshold was set at minimum of ≈90% sensitivity in detecting csPCa, aiming to optimize the highest possible specificity for a sensitivity not lower than ≈90%.


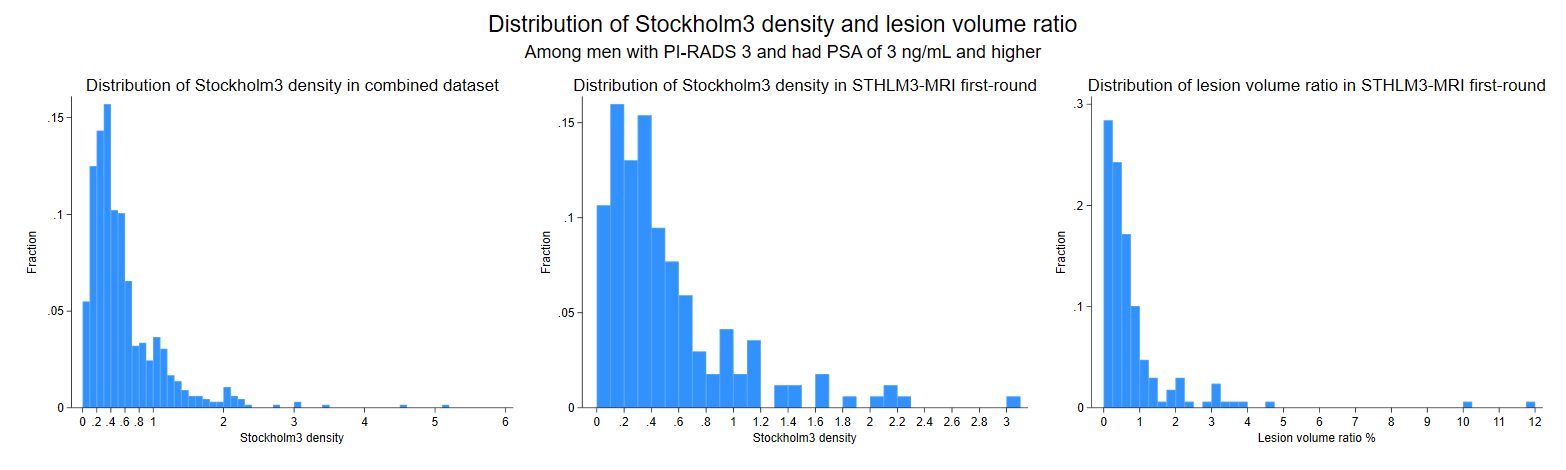


*eFigure (1): Histogram of the fraction distribution of the new tested biomarkers among men with PSA ≥3ng/mL and PI-RADS 3 in combined dataset (complete-case) and STHLM3-MRI first-round.*

| eTable (1): Selection of cut-off points of the biomarkers used to detect clinically significant prostate cancer^a^ among patients with PI-RADS 3 and PSA ≥ 3 | | | | |
| --- | --- | --- | --- | --- |
| Variable | Youden’s Index | AUC (95% CI)* | Liu’s Index | AUC (95% CI)* |
| **Combined dataset (complete-case)**^b^ | | | | |
| Age (in years) | 67 | 0.58 (0.54, 0.62) | 67 | 0.58 (0.54, 0.62) |
| Prostate volume (in mL) | 46 | 0.62 (0.58, 0.65) | 51 | 0.58 (0.54, 0.62) |
| PSA density (PSAD) | 0.12 | 0.58 (0.54, 0.62) | 0.12 | 0.58 (0.54, 0.62) |
| Stockholm3 | 15% | 0.60 (0.56, 0.64) | 19% | 0.59 (0.55, 0.63) |
| Stockholm3D | 0.39 | 0.63 (0.59, 0.67) | 0.39 | 0.63 (0.59, 0.67) |
| **Combined dataset (imputed)**^b^ | |  |  |  |
| Age (in years) | 67 | 0.57 (0.54, 0.60) | 67 | 0.57 (0.54, 0.60) |
| Prostate volume (in mL) | 40 | 0.59 (0.56, 0.62) | 51 | 0.59 (0.56, 0.62) |
| PSA density (PSAD) | 0.12 | 0.60 (0.57, 0.64) | 0.12 | 0.60 (0.57, 0.64) |
| Stockholm3 | 15% | 0.59 (0.55, 0.62) | 19% | 0.58 (0.54, 0.61) |
| Stockholm3D | 0.39 | 0.62 (0.59, 0.66) | 0.41 | 0.62 (0.59, 0.65) |
| Lesion volume (LV) (in mL) | 0.28 | 0.51 (0.48, 0.55) | 0.21 | 0.51 (0.47, 0.56) |
| Lesion volume ratio (LR) | 0.55% | 0.55 (0.51, 0.60) | 0.45% | 0.56 (0.51, 0.60) |
| **STHLM3-MRI RCT**  **first-round** | | | | |
| Age (in years) | 68 | 0.61 (0.53, 0.68) | 68 | 0.61 (0.53, 0.68) |
| Prostate volume (in mL) | 40 | 0.70 (0.63, 0.77) | 32.5 | 0.59 (0.52, 0.67) |
| PSAD | 0.14 | 0.62 (0.55, 0.69) | 0.12 | 0.61 (0.53, 0.69) |
| Stockholm3 | 15% | 0.66 (0.59, 0.73) | 15% | 0.66 (0.59, 0.73) |
| Stockholm3D | 0.37 | 0.69 (0.61, 0.76) | 0.39 | 0.69 (0.61, 0.76) |
| Lesion volume (LV) (in mL) | 0.25 | 0.58 (0.50, 0.66) | 0.25 | 0.58 (0.50, 0.66) |
| Lesion volume ratio (LR) | 0.6% | 0.64 (0.57, 0.72) | 0.6% | 0.64 (0.57, 0.72) |
| LR: MRI lesion volume ratio, LV: MRI lesion volume, PSA: prostate specific antigen, PSAD: prostate specific antigen density, PCa: prostate cancer, PI-RADS: Prostate Imaging-Reporting and Data System, Stockholm3D: Stockholm 3 density, DRE: digital rectal examination  * AUC calculated by running the logistic model with a variable dichotomized based on the cut-off point  ^a^ Clinically significant PCa is prostate cancer with ISUP grade ≥ 2  ^b^ Combined dataset using three datasets: STHLM3-MRI RCT first- and second-round screening, and Saint Göran’s hospital (Capio PCC) dataset  ^c^ The lesion volume (in mL) was computed using the volume of the largest suspicious lesion that facilitated the PI-RADS score of 3. The lesion volume ratio is calculated by dividing the MRI lesion volume (in mL) by the prostate volume (in mL) resulting in %. | | | | |

| eTable (2): Cut-point matrix for best-performing model based on AUC among men with PSA ≥ 3 ng/mL and PI-RADS 3 score | | | | | | |
| --- | --- | --- | --- | --- | --- | --- |
| **Combined dataset (complete-case)** | | | | |  |  |
|  | Age ≥ 67 | Prostate volume ≤ 45 mL | PSA density ≥ 0.12 | Stockholm 3 density ≥ 0.39 | Model Sensitivity | Model Specificity |
| Cut-point 1  (biopsy all) | No | No | No | No | 100% | 0% |
| Cut-point 2 | No | No | Yes | No | 96.51% | 19.10% |
| Cut-point 3 | No | No | No | Yes | 96.12% | 23.87% |
| Cut-point 4 | No | Yes | No | No | 94.96% | 26.38% |
| Cut-point 5 | Yes | No | No | No | 89.53% | 36.18% |
| Cut-point 6 | No | No | Yes | Yes | 75.97% | 52.01% |
| Cut-point 7 | No | Yes | Yes | No | 73.64% | 56.78% |
| Cut-point 8 | Yes | No | Yes | No | 70.93% | 58.29% |
| Cut-point 9 | No | Yes | No | Yes | 68.22% | 60.05% |
| Cut-point 10 | Yes | No | No | Yes | 60.08% | 64.82% |
| Cut-point 11 | Yes | Yes | No | No | 55.81% | 70.35% |
| Cut-point 12 | No | Yes | Yes | Yes | 54.26% | 72.86% |
| Cut-point 13 | Yes | No | Yes | Yes | 31.78% | 87.19% |
| Cut-point 14 | Yes | Yes | Yes | No | 27.52% | 88.44% |
| Cut-point 15 | Yes | Yes | No | Yes | 25.97% | 90.45% |
| Cut-point 16 | Yes | Yes | Yes | Yes | 17.44% | 93.22% |
| Biopsy none |  |  |  |  | 0% | 100% |
| **STHLM3-MRI clinical trial first-round** | | | | | | |
|  | Age ≥ 68 | Prostate volume ≤ 40 mL | Stockholm 3 density ≥ 0.37 | MRI lesion volume ratio ≥ 0.6% | Model Sensitivity | Model Specificity |
| Cut-point 1 (biopsy all) | No | No | No | No | 100% | 0% |
| Cut-point 2 | No | No | Yes | No | 100% | 23.36% |
| Cut-point 3 | No | No | No | Yes | 100% | 31.78% |
| Cut-point 4 | No | Yes | No | No | 100% | 42.99% |
| Cut-point 5 | Yes | No | No | No | 98.39% | 50.47% |
| Cut-point 6 | No | No | Yes | Yes | 85.48% | 65.42% |
| Cut-point 7 | No | Yes | Yes | No | 79.03% | 67.29% |
| Cut-point 8 | No | Yes | No | Yes | 66.13% | 72.90% |
| Cut-point 9 | Yes | No | Yes | No | 59.68% | 75.70% |
| Cut-point 10 | Yes | No | No | Yes | 56.45% | 79.44% |
| Cut-point 11 | Yes | Yes | No | No | 53.23% | 81.31% |
| Cut-point 12 | No | Yes | Yes | Yes | 53.23% | 84.11% |
| Cut-point 13 | Yes | No | Yes | Yes | 32.26% | 93.46% |
| Cut-point 14 | Yes | Yes | Yes | No | 30.65% | 95.33% |
| Cut-point 15 | Yes | Yes | No | Yes | 19.35% | 99.07% |
| Cut-point 16 | Yes | Yes | Yes | Yes | 16.13% | 99.07% |
| Biopsy none |  |  |  |  | 0% | 100% |

| eTable (3): Demographic and clinical characteristics of men with PI-RADS 3 score, and had PSA of 3 ng/mL and higher, or PSA of 1.5-3 ng/mL and Stockholm3 of 11% |
| --- |

|  | STHLM3-MRI First-round | STLHM3-MRI Second-round | Capio PCC | Total |
| --- | --- | --- | --- | --- |
|  | n=252 (28.47%) | n=100 (11.30%) | n=794 (69%) | n=1146 (100%) |
| Age in years, mean (SD) | 65 (6.17) | 66 (6.97) | 66 (7.04) | 66 (6.85) |
| PSA (ng/mL), median (IQR) | 3.5 (2.6, 4.7) | 3.5 (2.9, 4.7) | 4.9 (3.4, 7.6) | 4.3 (3.1, 6.8) |
| Not reported | 0 (0%) | 0 (0%) | 42 (5%) | 42 (4%) |
| Prostate volume (in mL), median (IQR) | 37 (28, 52) | 43.5 (32, 52) | 44 (31, 59) | 42 (60, 57) |
| Not reported | 0 (0%) | 0 (0%) | 14 (2%) | 14 (1%) |
| PSA Density, median (IQR) | 0.09 (0.07, 0.13) | 0.08 (0.06, 0.11) | 0.11 (0.08, 0.17) | 0.10 (0.07, 0.15) |
| Not reported | 0 (0%) | 0 (0%) | 56 (7%) | 56 (5%) |
| Stockholm3 test score, median (IQR) | 15 (11, 22) | 14 (11, 21) | 19 (14, 28) | 17 (12, 26) |
| Not reported | 0 (0%) | 0 (0%) | 231 (29%) | 231 (20%) |
| Stockholm 3 Density, median (IQR) | 0.41 (0.26, 0.65) | 0.34 (0.24, 0.51) | 0.47 (0.30, 0.73) | 0.42 (0.28, 0.68) |
| Not reported | 0 (0%) | 0 (0%) | 233 (29%) | 233 (20%) |
| MRI Lesion Volume (in mL), median (IQR) | 0.17 (0.10, 0.33) | 0.20 (0.10, 0.33) | N/A | N/A |
| Not reported | 0 (0%) | 0 (0%) | 794 (100%) | 794 (69%) |
| MRI Lesion Ratio, median (IQR) | 0.45% (0.24%, 0.93%) | 0.45% (0.22%, 0.93%) | N/A | N/A |
| Not reported | 0 (0%) | 0 (0%) | 794 (100%) | 794 (69%) |
| Suspicious digital rectal examination finding, n (%) |  |  |  |  |
| No | 154 (61%) | 28 (28%) | N/A | N/A |
| Yes | 93 (37%) | 62 (62%) | N/A | N/A |
| Not reported | 5 (2%) | 10 (10%) | 794 (100%) | 809 (71%) |
| Lowest Mean Apparent Diffusion Coefficient (in mm^2^/s) median (IQR) | 0.947x10^-3^ (0.836x10^-3^, 1.029x10^-3^) | 0.922x10^-3^ (0.845x10^-3^, 0.980x10^-3^) | N/A | N/A |
| Not reported | 16 (9%) | 4 (6%) | 794 (100%) | 814 (71%) |
| Diffusion-Weighted Imaging |  |  |  |  |
| 2 | 1 (0.4%) | 0 (0%) |  |  |
| 3 | 170 (71%) | 75 (77%) | N/A | N/A |
| 4 | 65 (27%) | 22 (22%) | N/A | N/A |
| 5 | 3 (1%) | 1 (1%) | N/A | N/A |
| Not reported | 13 (5%) | 2 (3%) | 794 (100%) | 809 (71%) |
| ISUP, n (%) |  |  |  |  |
| Benign | 110 (44%) | 40 (40%) | 361 (45%) | 511 (45%) |
| ISUP 1 (GG 6) | 48 (19%) | 18 (18%) | 112 (14%) | 178 (16%) |
| ISUP 2 | 81 (32%) | 30 (30%) | 265 (33%) | 376 (33%) |
| ISUP 3 | 6 (2%) | 3 (3%) | 31 (4%) | 40 (3%) |
| ISUP 4 | 3 (1%) | 1 (1%) | 13 (2%) | 17 (1%) |
| ISUP 5 | 4 (2%) | 8 (8%) | 12 (2%) | 24 (2%) |
| Clinically significant prostate cancer (ISUP 2+), n (%) |  |  |  |  |
| No | 158 (63%) | 58 (58%) | 473 (60%) | 689 (60%) |
| Yes | 94 (37%) | 42 (42%) | 321 (40%) | 457 (40%) |
| High-grade prostate cancer (ISUP 3+), n (%) |  |  |  |  |
| No | 239 (95%) | 88 (88%) | 738 (93%) | 1065 (93%) |
| Yes | 13 (5%) | 12 (12%) | 56 (7%) | 81 (7%) |
| GG: Gleason grade, PCC: prostate cancer center, PSA: prostate specific antigen, ISUP: International Society of Urological Pathology, ng: nanogram, mL: milliliter, IQR: interquartile range | | | | |


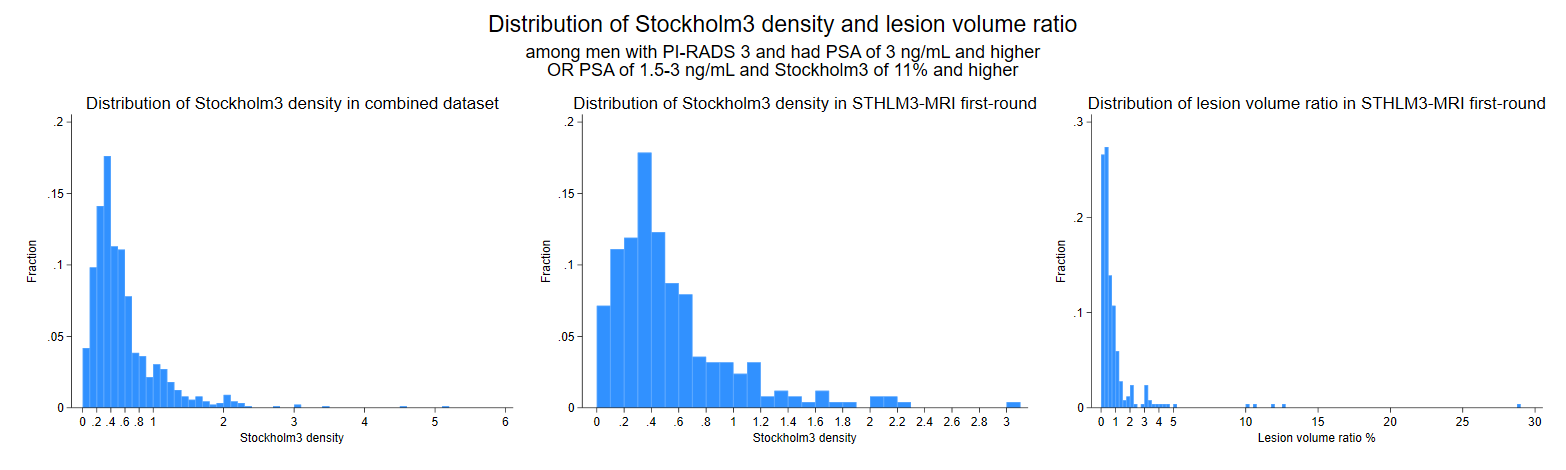


*eFigure (2): Histogram of the fraction distribution of the new tested biomarkers among men with PI-RADS 3 in combined dataset (complete-case) and STHLM3-MRI first-round.*

| eTable (4): Predictive capacity to detect clinically significant prostate cancer^a^ among patients with PI-RADS 3 in the three cohorts | | | | | | | |
| --- | --- | --- | --- | --- | --- | --- | --- |
| Models | STHLM3-MRI RCT  first-round screening | | STHLM3-MRI RCT  second-round screening | Capio PCC | | Combined dataset  (complete-case) | Combined dataset  (imputed) |
| **Individual continuous variables** | AUC (95% CI) | | AUC (95% CI) | AUC (95% CI) | | AUC (95% CI) | AUC (95% CI) |
| Age | 0.55 (0.48, 0.62) | | 0.56 (0.49, 0.67) | 0.54 (0.49, 0.59) | | 0.55 (0.50, 0.59) | 0.56 (0.52, 0.59) |
| Prostate volume | 0.64 (0.57, 0.71) | | 0.56 (0.47, 0.67) | 0.60 (0.55, 0.64) | | 0.60 (0.57, 0.65) | 0.62 (0.59, 0.65) |
| PSA density (PSAD) | 0.60 (0.52, 0.67) | | 0.58 (0.48, 0.69) | 0.59 (0.54, 0.64) | | 0.59 (0.55, 0.63) | 0.62 (0.58, 0.65) |
| Stockholm 3 | 0.64 (0.57, 0.71) | | 0.61 (0.49, 0.72) | 0.60 (0.55, 0.65) | | 0.61 (0.58, 0.65) | 0.60 (0.57, 0.64) |
| Stockholm 3D | 0.68 (0.62, 0.75) | | 0.60 (0.48, 0.71) | 0.63 (0.58, 0.67) | | 0.64 (0.60, 0.67) | 0.65 (0.61, 0.68) |
| Lesion Volume (LV)^b^ | 0.48 (0.39, 0.55) | | 0.50 (0.37, 0.59) | N/A | | N/A | 0.51 (0.46, 0.55) |
| Lesion Ratio (LR)^b^ | 0.58 (0.40, 0.67) | | 0.52 (0.41, 0.62) | N/A | | N/A | 0.55 (0.47, 0.62) |
| Digital Rectal Examination (DRE) | 0.58 (0.52, 0.65) | | 0.60 (0.51, 0.69) | N/A | | N/A | 0.52 (0.48, 0.56) |
| Models | | STHLM3-MRI RCT  first-round screening | | | Combined dataset  (complete-case) | | Combined dataset  (imputed) |
| **Risk calculators** | | AUC (95% CI) | | | AUC (95% CI) | | AUC (95% CI) |
| the Prostate Biopsy Collaborative Group (PBCG) model | | 0.68 (0.61, 0.74) | | | N/A | | 0.64 (0.61, 0.67) |
| Mehralivand et al. model | | 0.68 (0.61, 0.75) | | | N/A | | 0.66 (0.63, 0.70) |
| Mount Sinai Prebiopsy Risk Calculator (MSP-RC) | | 0.63 (0.56, 0.70) | | | N/A | | 0.62 (0.58, 0.65) |
| Prospective Loyola University multiparametric MRI (PLUM) | | 0.67 (0.60, 0.73) | | | N/A | | 0.65 (0.62, 0.69) |
| European Randomised Study of Screening for Prostate Cancer risk-calculator (ERSPC-RC) | | 0.54 (0.47, 0.62) | | | N/A | | 0.56 (0.52, 0.61) |
| van Leeuwen et al. Model | | 0.64 (0.56, 0.70) | | | N/A | | 0.66 (0.62, 0.69) |
| Perez et al. Model | | 0.61 (0.54, 0.68) | | | N/A | | 0.58 (0.53, 0.62) |
| LR: MRI lesion volume ratio, LV: MRI lesion volume, PSA: prostate specific antigen, PSAD: prostate specific antigen density, PCa: prostate cancer, PCC: prostate cancer center PI-RADS: Prostate Imaging-Reporting and Data System, Stockholm3D: Stockholm3 density.  ^a^ Clinically significant PCa is prostate cancer with ISUP grade ≥ 2  ^b^ The lesion volume (in mL) was computed using the volume of the suspicious lesion that facilitated the PI-RADS score of 3. The lesion volume ratio is calculated by dividing the MRI lesion volume (in mL) by the prostate volume (in mL)  ^c^ Dichotomized variables were generated using Youden’s Index and/or Liu’s Index. The choice of the cut-off point considered the AUC and sensitivity/specificity performance  * PSAD not significant in the model  ^♦^ LR not significant in the model | | | | | | | |

| eTable (5): Predictive capacity to detect clinically significant prostate cancer^a^ among patients with PI-RADS 3 in the three cohorts | | | |
| --- | --- | --- | --- |
| Models* | Combined dataset  (complete-case) | STHLM3-MRI RCT  first-round screening |  |
| **Prediction tests based on probability^c^** | AUC (95% CI) | AUC (95% CI) |  |
| Age and PSAD | 0.59 (0.55, 0.63) | 0.60 (0.53, 0.68) |  |
| PSAD and Stockholm 3 | 0.62 (0.59, 0.66) | 0.65 (0.58, 0.72) |  |
| Age, prostate volume, and Stockholm 3 | 0.63 (0.59, 0.67) | 0.68 (0.61, 0.75) |  |
| Age, prostate volume, PSAD, and Stockholm 3D | 0.63 (0.60, 0.67) | 0.67 (0.61, 0.74) |  |
| Age, PSAD, and LV^d^ | N/A | 0.60 (0.53, 0.68) |  |
| Age, prostate volume, Stockholm 3D, and LV^d^ | N/A | 0.67 (0.61, 0.74) |  |
| Age, PSAD, and LR | N/A | 0.60 (0.53, 0.68) |  |
| Age, prostate volume, Stockholm 3D, and LR | N/A | 0.67 (0.61, 0.74) |  |
| Age, prostate volume, Stockholm 3, LR | N/A | 0.68 (0.61, 0.75) |  |
| **Model based on dichotomized variables^e^** | AUC (95% CI) | AUC (95% CI) |  |
| Age, PSAD, and Stockholm 3 | 0.64 (0.60, 0.68) | 0.67 (0.61, 0.74) |  |
| Age, prostate volume, and Stockholm 3 | 0.66 (0.62, 0.70) | 0.71 (0.65, 0.78) |  |
| Age, prostate volume, PSAD, and Stockholm3D | 0.67 (0.64, 0.71) | 0.74 (0.68, 0.80)^f^ |  |
| Age, prostate volume, Stockholm 3D, LVR | N/A | 0.75 (0.69, 0.81) |  |
| Age, prostate volume, Stockholm 3D, LVR, DRE | N/A | 0.77 (0.71, 0.83) |  |
| Age, PSAD, and LV^d^ | N/A | 0.66 (0.59, 0.72) |  |
| Age, prostate volume, Stockholm 3D, and LV^d^ | N/A | 0.74 (0.68, 0.80) |  |
| Age, PSAD, and LR | N/A | 0.67 (0.61, 0.74) |  |
| Age, prostate volume, Stockholm 3, LR | N/A | 0.74 (0.68, 0.80) |  |
| LR: MRI lesion ratio, LV: MRI lesion volume, PSA: prostate specific antigen, PSAD: prostate specific antigen density, PCa: prostate cancer, PCC: prostate cancer center PI-RADS: Prostate Imaging-Reporting and Data System, Stockholm3D: Stockholm3 density, DRE: digital rectal examination.  ^a^ Clinically significant PCa is prostate cancer with ISUP grade ≥ 2  ^b^ The lesion volume (in mL) was computed using the volume of the largest suspicious lesion that facilitated the PI-RADS score of 3. The lesion volume ratio is calculated by dividing the MRI lesion volume (in mL) by the prostate volume (in mL) resulting in %.  ^c^ The prediction tests have values between 0 to 1 based on the probability of having clinically significant prostate cancer based on the continuous variables used in the model  ^d^ LV was not statistically significant in the logistic model  ^e^ Dichotomized variables were generated using Youden’s Index and/or Liu’s Index. The choice of the cut-off point considered the AUC and sensitivity/specificity performance  ^f^ PSAD was not statistically significant in the logistic model | | |  |

| eTable (6): Selection of cut-off points of the biomarkers used to detect clinically significant prostate cancer^a^ among patients with PI-RADS 3 | | | | |
| --- | --- | --- | --- | --- |
| Variable | Youden’s Index | AUC (95% CI)* | Liu’s Index | AUC (95% CI)* |
| **Combined dataset (complete-case)**^b^ | | | | |
| Age (in years) | 69 | 0.56 (0.52, 0.60) | 66 | 0.56 (0.53, 0.60) |
| Prostate volume (in mL) | 46 | 0.59 (0.55, 0.62) | 42 | 0.57 (0.54, 0.61) |
| PSA density (PSAD) | 0.12 | 0.58 (0.54, 0.61) | 0.10 | 0.57 (0.53, 0.60) |
| Stockholm3 | 14% | 0.59 (0.55, 0.62) | 19% | 0.58 (0.54, 0.62) |
| Stockholm3D | 0.39 | 0.62 (0.58, 0.66) | 0.39 | 0.62 (0.58, 0.66) |
| **Combined dataset (imputed)**^b^ | |  |  |  |
| Age (in years) | 69 | 0.56 (0.53, 0.59) | 66.5 | 0.56 (0.53, 0.58) |
| Prostate volume (in mL) | 41.5 | 0.58 (0.55, 0.61) | 42 | 0.58 (0.55, 0.61) |
| PSA density (PSAD) | 0.12 | 0.60 (0.57, 0.63) | 0.12 | 0.60 (0.57, 0.63) |
| Stockholm3 | 18% | 0.57 (0.54, 0.60) | 19% | 0.58 (0.54, 0.61) |
| Stockholm3D | 0.39 | 0.62 (0.59, 0.65) | 0.42 | 0.61 (0.58, 0.64) |
| Lesion volume (LV) (in mL) | 0.32 | 0.51 (0.48, 0.55) | 0.20 | 0.51 (0.48, 0.54) |
| Lesion ratio (LR) | 0.48% | 0.54 (0.50, 0.58) | 0.44% | 0.54 (0.50, 0.58) |
| **STHLM3-MRI RCT**  **first-round screening**  **(baseline)** | | | | |
| Age (in years) | 69 | 0.58 (0.52, 0.64) | 68 | 0.58 (0.51, 0.64) |
| Prostate volume (in mL) | 38 | 0.66 (0.60, 0.72) | 33 | 0.58 (0.51, 0.64) |
| PSAD | 0.14 | 0.59 (0.53, 0.64) | 0.09 | 0.58 (0.51, 0.64) |
| Stockholm3 | 13% | 0.61 (0.55, 0.67) | 14% | 0.62 (0.56, 0.68) |
| Stockholm3D | 0.37 | 0.67 (0.61, 0.73) | 0.37 | 0.67 (0.61, 0.73) |
| Lesion volume (LV) (in mL) | 0.49 | 0.55 (0.48, 0.61) | 0.47 | 0.55 (0.48, 0.61) |
| Lesion ratio (LR) | 0.6% | 0.60 (0.54, 0.66) | 0.6% | 0.60 (0.54, 0.66) |
| LR: MRI lesion ratio, LV: MRI lesion volume PSA: prostate specific antigen, PSAD: prostate specific antigen density, PCa: prostate cancer, PI-RADS: Prostate Imaging-Reporting and Data System, Stockholm 3D: Stockholm 3 density  * AUC calculated by running the logistic model with a variable dichotomized based on the cut-off point  ^a^ Clinically significant PCa is prostate cancer with ISUP grade ≥ 2  ^b^ Combined dataset using three datasets: STHLM3-MRI RCT first- and second-round screening, and Saint Göran’s hospital dataset  ^c^ Development subset includes 75% of the combined dataset randomly allocated. 10 subset repeats were created and an average was computed  ^c^ The lesion volume (in mL) was computed using the volume of the largest suspicious lesion that facilitated the PI-RADS score of 3 extracted from the MRI. The lesion ratio is calculated by dividing the MRI lesion volume (in mL) by the prostate volume (in mL) | | | | |

| eTable (7): Cut-point matrix for best-performing model based on AUC | | | | | | | | | | | |
| --- | --- | --- | --- | --- | --- | --- | --- | --- | --- | --- | --- |
| **Combined dataset (complete-case)** | | | | | | | | | |  |  |
|  | | Age ≥ 67 | | Prostate volume < 45 mL | | PSA density ≥ 0.12 | | Stockholm 3 density ≥ 0.39 | | Model Sensitivity | Model Specificity |
| Cut-point 1  (biopsy all) | | No | | No | | No | | No | | 100% | 0% |
| Cut-point 2 | | No | | No | | Yes | | No | | 97.04% | 14.81% |
| Cut-point 3 | | No | | Yes | | No | | No | | 96.75% | 18.28% |
| Cut-point 4 | | Yes | | No | | No | | No | | 89.94% | 30.35% |
| Cut-point 5 | | No | | No | | No | | Yes | | 78.40% | 45.16% |
| Cut-point 6 | | No | | Yes | | Yes | | No | | 77.51% | 46.98% |
| Cut-point 7 | | Yes | | No | | Yes | | No | | 75.44% | 48.26% |
| Cut-point 8 | | No | | No | | Yes | | Yes | | 73.37% | 49.91% |
| Cut-point 9 | | Yes | | Yes | | No | | No | | 71.89% | 53.38% |
| Cut-point 10 | | No | | Yes | | No | | Yes | | 68.64% | 57.95% |
| Cut-point 11 | | Yes | | No | | No | | Yes | | 55.33% | 67.28% |
| Cut-point 12 | | Yes | | Yes | | Yes | | No | | 50.89% | 72.03% |
| Cut-point 13 | | No | | Yes | | Yes | | Yes | | 49.70% | 73.49% |
| Cut-point 14 | | Yes | | No | | Yes | | Yes | | 32.54% | 84.10% |
| Cut-point 15 | | Yes | | Yes | | No | | Yes | | 28.70% | 85.19% |
| Cut-point 16 | | Yes | | Yes | | Yes | | Yes | | 16.27% | 94.33% |
| Biopsy none | |  | |  | |  | |  | | 0% | 100% |
| **STHLM3-MRI clinical trial first-round** | | | | | | | | | | | |
|  | Age ≥ 69 | | Prostate volume < 38 mL | | Stockholm 3 density ≥ 0.37 | | MRI lesion ratio ≥ 0.6% | | DRE suspicious finding | Model Sensitivity | Model Specificity |
| Cut-point 1 (biopsy all) | No | | No | | No | | No | | No | 100% | 0% |
| Cut-point 2 | No | | No | | No | | No | | Yes | 98.91% | 16.77% |
| Cut-point 3 | No | | No | | No | | Yes | | No | 97.83% | 22.58% |
| Cut-point 4 | No | | No | | Yes | | No | | No | 96.74% | 28.39 |
| Cut-point 5 | No | | Yes | | No | | No | | No | 96.74% | 32.90% |
| Cut-point 6 | Yes | | No | | No | | No | | No | 96.74% | 36.77% |
| Cut-point 7 | No | | No | | No | | Yes | | Yes | 92.39% | 43.23% |
| Cut-point 8 | No | | No | | Yes | | No | | Yes | 90.22% | 47.10% |
| Cut-point 9 | No | | No | | Yes | | Yes | | No | 89.13% | 49.03% |
| Cut-point 10 | No | | Yes | | No | | No | | Yes | 85.87% | 50.32% |
| Cut-point 11 | No | | Yes | | No | | Yes | | No | 84.78% | 52.90% |
| Cut-point 12 | Yes | | No | | No | | No | | Yes | 80.43% | 54.84% |
| Cut-point 13 | Yes | | No | | No | | Yes | | No | 78.26% | 60.00% |
| Cut-point 14 | No | | Yes | | Yes | | No | | No | 76.09% | 61.94% |
| Cut-point 15 | Yes | | No | | Yes | | No | | No | 68.48% | 68.39% |
| Cut-point 16 | Yes | | Yes | | No | | No | | No | 65.22% | 71.61% |
| Cut-point 17 | No | | No | | Yes | | Yes | | Yes | 65.22% | 72.26% |
| Cut-point 18 | Yes | | No | | No | | Yes | | Yes | 63.04% | 72.26% |
| Cut-point 19 | No | | Yes | | Yes | | No | | Yes | 63.04% | 72.90% |
| Cut-point 20 | No | | Yes | | No | | Yes | | Yes | 54.35% | 78.06% |
| Cut-point 21 | Yes | | No | | Yes | | No | | Yes | 44.57% | 86.45% |
| Cut-point 22 | Yes | | No | | Yes | | Yes | | No | 41.30% | 87.74% |
| Cut-point 23 | Yes | | Yes | | No | | Yes | | No | 41.30% | 88.39% |
| Cut-point 24 | Yes | | Yes | | Yes | | No | | No | 40.22% | 88.39% |
| Cut-point 25 | No | | Yes | | Yes | | Yes | | Yes | 33.70% | 92.26% |
| Cut-point 26 | Yes | | No | | Yes | | Yes | | Yes | 21.74% | 96.13% |
| Cut-point 27 | Yes | | Yes | | Yes | | No | | Yes | 20.65% | 97.42% |
| Cut-point 28 | Yes | | Yes | | Yes | | Yes | | No | 14.13% | 97.42% |
| Cut-point 29 | Yes | | Yes | | Yes | | Yes | | Yes | 6.52% | 100% |
| Biopsy none |  | |  | |  | |  | |  | 0% | 100% |

| eTable (8): Predictive table based on best performing models and special considerations for PSA density and Stockholm 3 test of men with PI-RADS 3 score on MRI | | | | | |
| --- | --- | --- | --- | --- | --- |
| **Combined dataset (complete-case)** | | | | | |
| Consideration^a^, n (%; 95%CI) | Biopsies performed | Detected csPCa^b^ | Detected ISUP 3+ | Detected GG6 | Avoided unnecessary biopsies (GG6 and lower) |
| Biopsy all | 885 (100%; 100%, 100%) | 338 (100%; 99%, 100%) | 56 (100%; 94%, 100%) | 146 (100%; 98%, 100%) | 0 (0%; 0%, 1%) |
| PSAD ≥ 0.075 | 621 (70%; 67%, 73%) | 260 (77%; 72%, 81%) | 42 (75%; 62%, 86%) | 108 (74%; 66%, 81%) | 186 (34%; 30%, 38%) |
| PSAD ≥ 0.10 | 418 (47%; 44%, 51%) | 185 (55%; 50%, 60%) | 30 (54%; 40%, 67%) | 70 (48%; 40%, 56%) | 314 (57%; 53%, 62%) |
| PSAD ≥ 0.15 | 192 (22%; 19%, 25%) | 97 (29%; 24%, 34%) | 20 (36%; 23%, 50%) | 29 (20%; 14%, 27%) | 452 (83%; 79%, 86%) |
| PSAD ≥ 0.20 | 94 (11%; 9%, 13%) | 56 (17%; 13%, 21%) | 11 (20%; 10%, 32%) | 13 (9%; 5%, 15%) | 509 (93%; 91%, 95%) |
| Stockholm3 ≥ 11% | 787 (89%; 87%, 91%) | 317 (94%; 91%, 96%) | 51 (91%; 80%, 97%) | 129 (88%; 82%, 93%) | 77 (14%; 11%, 17%) |
| Stockholm3 ≥ 13% | 653 (74%; 71%, 77%) | 280 (83%; 78%, 88%) | 49 (88%; 76%, 95%) | 106 (73%; 65%, 80%) | 174 (32%; 28%, 36%) |
| Stockholm3 ≥ 15% | 561 (63%; 60%, 67%) | 251 (74%; 69%, 79%) | 47 (84%; 72%, 92%) | 95 (65%; 57%, 73%) | 237 (43%; 39%, 48%) |
| Best performing model* | 685 (77%; 75%, 80%) | 304 (90%; 86%, 93%) | 53 (95%; 85%, 99%) | 111 (76%; 68%, 83%) | 166 (30%; 27%, 34%) |
| Biopsy none | 0 (0%; 0%, 0%) | 0 (0%; 0%, 1%^♦^) | 0 (0%; 0%; 6%^♦^) | 0 (0%; 0%, 2%^♦^) | 547 (100%; 99%^♦^, 100%) |
| **STHLM3-MRI RCT First-round** | | | | | |
| Consideration^a^, n (%) | Biopsies performed | Detected csPCa^b^ | Detected ISUP 3+ | Detected GG6 | Avoided unnecessary biopsies (GG6 and lower) |
| Biopsy all | 252 (100%; 99%^♦^, 100%) | 94 (100%; 96%, 100%) | 13 (100%; 75%, 100%) | 48 (100%; 93%, 100%) | 0 (0%; 0%, 2%) |
| PSAD ≥ 0.075 | 165 (65%; 59%, 71%) | 68 (72%; 62%, 81%) | 10 (77%; 46%, 95%) | 35 (73%; 58%, 85%) | 61 (39%; 31%, 47%) |
| PSAD ≥ 0.10 | 111 (44%; 38%, 50%) | 48 (51%; 41%, 62%) | 8 (62%; 32%, 86%) | 22 (46%; 31%, 61%) | 95 (60%; 52%, 68%) |
| PSAD ≥ 0.15 | 43 (17%; 13%, 22%) | 24 (26%; 17%, 36%) | 6 (46%; 19%, 75%) | 6 (13%; 5%, 25%) | 139 (88%; 82%, 93%) |
| PSAD ≥ 0.20 | 17 (7%; 4%, 11%) | 11 (12%; 6%, 20%) | 4 (31%; 9%, 61%) | 3 (6%; 1%, 17%) | 152 (96%; 92%, 99%) |
| Stockholm3 ≥ 11% | 202 (80%; 75%, 85%) | 83 (88%; 80%, 94%) | 9 (69%; 39%, 91%) | 38 (79%; 65%, 90%) | 39 (25%; 18%, 32%) |
| Stockholm3 ≥ 13% | 159 (63%; 57%, 69%) | 72 (77%; 67%, 85%) | 9 (69%; 39%, 91%) | 27 (56%; 41%, 71%) | 71 (45%; 49%, 65%) |
| Stockholm3 ≥ 15% | 127 (50%; 44%, 57%) | 60 (64%; 53%, 73%) | 8 (62%; 32%, 86%) | 22 (46%; 31%, 61%) | 91 (58%; 49%, 65%) |
| Best performing model^#^ | 170 (67%; 61%, 73%) | 85 (90%; 83%, 96%) | 9 (69%; 39%, 91%) | 31 (65%; 49%, 78%) | 73 (46%; 38%, 54%) |
| Biopsy none | 0 (0%; 0%, 1%^♦^) | 0 (0%; 0%, 4%^♦^) | 0 (0%; 0%, 25%^♦^) | 0 (0%; 0%, 7%^♦^) | 158 (100%; 98%^♦^, 100%) |
| GG: Gleason grade, LR: MRI lesion ratio, LV: MRI lesion volume PSA: prostate specific antigen, PSAD: prostate specific antigen density, PCa: prostate cancer, PI-RADS: Prostate Imaging-Reporting and Data System, Stockholm3D: Stockholm3 density  * Best performing model consist of prostate volume, age, Stockholm3D, and PSA density  # Best performing model consist of prostate volume, age, Stockholm3D, MRI lesion volume ratio, digital rectal examination  ^a^ models compare to screening all patients with biopsies. Each column is compared separately.  ^b^ Significant PCa is prostate cancer with ISUP grade ≥ 2  ^♦^ Based on statistical computation, and might not be possible estimates in real-world setting | | | | | |

eTable (9): Outcomes of imputation

| Variables | STHLM3-MRI RCT  first-round screening | STHLM3-MRI RCT  second-round screening | Capio PCC | Combined dataset |
| --- | --- | --- | --- | --- |
| Number of observations imputed |  |  |  |  |
| Prostate-specific antigen (PSA) | 0 | 0 | 42 | 42 |
| Prostate volume | 0 | 0 | 14 | 14 |
| Stockholm3 test | 0 | 0 | 231 | 231 |
| Lesion Volume (LV) | 0 | 0 | 794 | 794 |
| Digital Rectal Examination (DRE) | 5 | 10 | 794 | 809 |
| Characteristics following imputation | | | | |
| Prostate-specific antigen (PSA), mean (SE) | 4.18 (0.16) | 3.96 (0.17) | 6.63 (0.23) | 5.86 (0.17) |
| Prostate volume, mean (SE) | 43 (1.39) | 46 (1.70) | 50 (0.99) | 48 (0.77) |
| Stockholm3 test, mean (SE) | 18 (0.72) | 17 (0.93) | 23.46 (0.57) | 21.7 (0.44) |
| Lesion Volume (LV), mean (SE) | 0.40 (0.07) | 0.29 (0.04) | 0.40 (0.06) | 0.39 (0.04) |
| Digital Rectal Examination (DRE), % (SE) |  |  |  |  |
| No | 62% (0.03) | 34% (0.05) | 57% (0.04) | 56% (0.03) |
| Yes | 38% (0.03) | 66% (0.05) | 43% (0.04) | 44% (0.03) |
| Imputation relative efficiency (50 imputations) | Relative efficiency | | | |
| Prostate-specific antigen (PSA) | 0.99647 | | | |
| Prostate volume | 0.998671 | | | |
| Stockholm3 test | 0.995086 | | | |
| Lesion Volume (LV) | 0.990391 | | | |
| Digital Rectal Examination (DRE) | 0.991522 | | | |


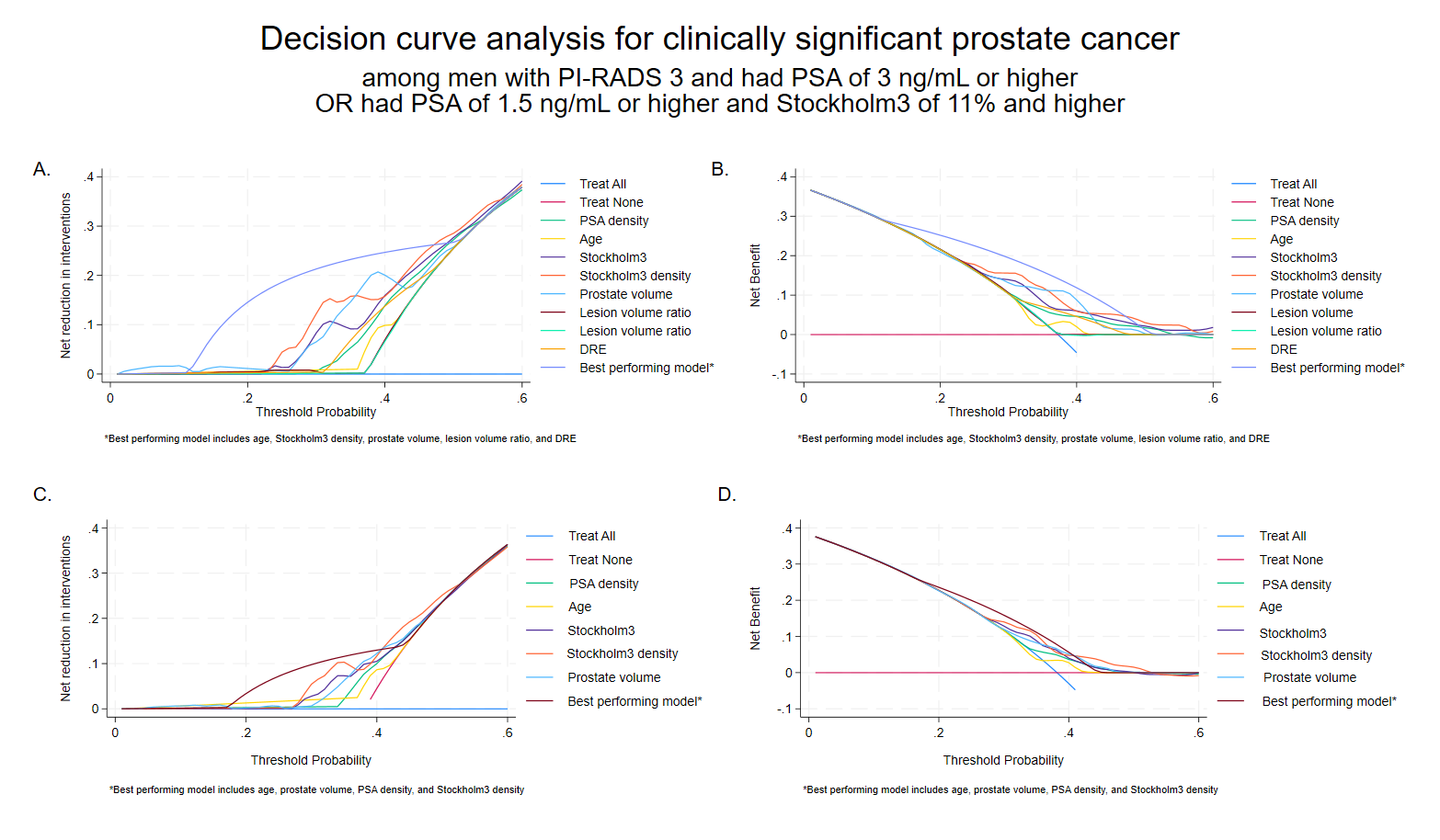


*eFigure (3): Decision curve analysis of individual biomarkers and best performing models in combined dataset (complete-case) and STHLM3-MRI first-round for all men with PI-RADS 3. A. Shows net reductions in the combined dataset (complete-case), B. Shows net reductions in STHLM3-MRI first-round, C. Shows net benefit in the combined dataset (complete-case), D. Shows net benefit in STHLM3-MRI first-round*


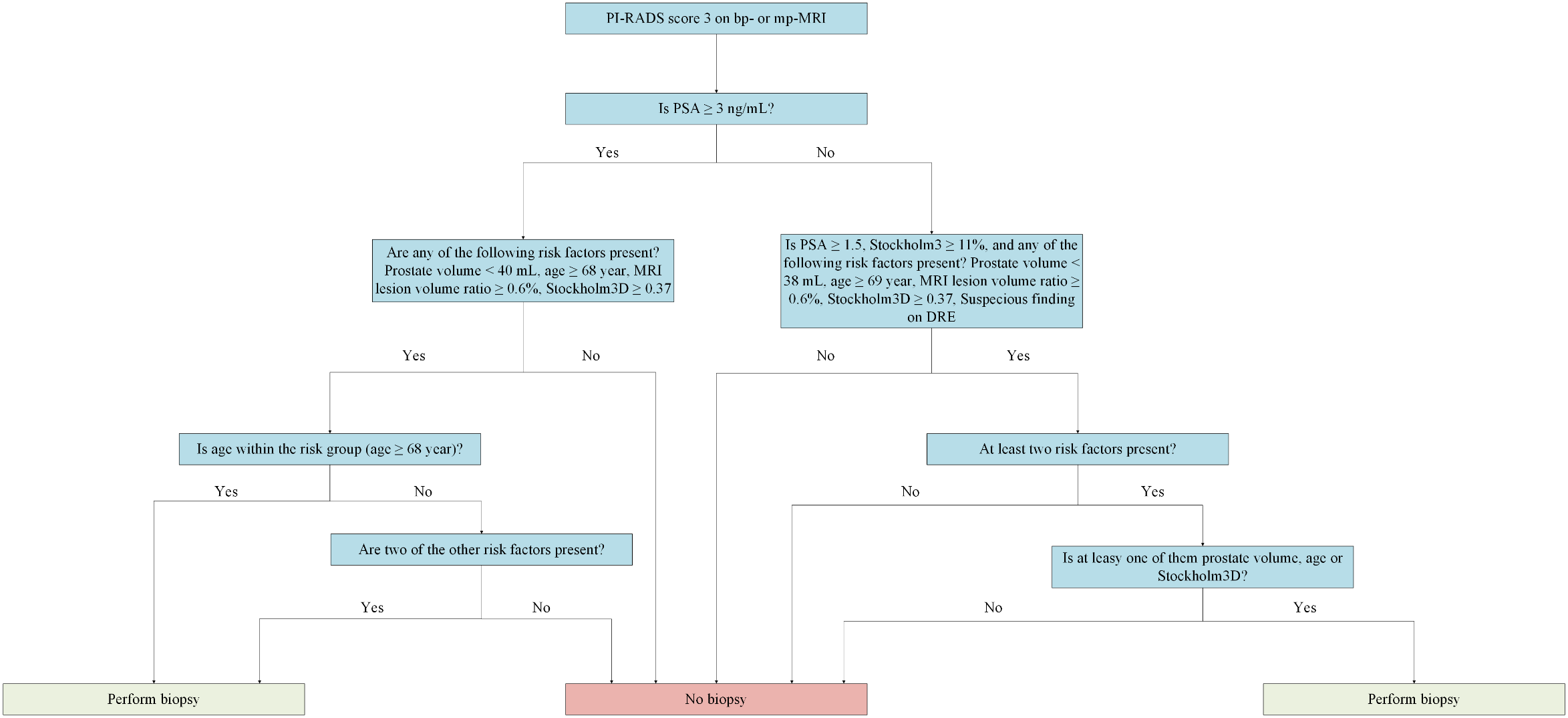


*eFigure (4): Operationalization of best performing models in a clinical decision matrix for guidance in biopsy decision among men with PI-RADS score 3 based on best performing models.*

**References:**

1. Nordström T, Discacciati A, Bergman M, et al. Prostate cancer screening using a combination of risk-prediction, MRI, and targeted prostate biopsies (STHLM3-MRI): a prospective, population-based, randomised, open-label, non-inferiority trial. *The Lancet Oncology*. 2021/09/01/ 2021;22(9):1240-1249. doi:<https://doi.org/10.1016/S1470-2045(21)00348-X>

2. Nordström T, Annerstedt M, Glaessgen A, et al. Repeated Prostate Cancer Screening Using Prostate-Specific Antigen Testing and Magnetic Resonance Imaging: A Secondary Analysis of the STHLM3-MRI Randomized Clinical Trial. *JAMA network open*. 2024;7(2):e2354577-e2354577. doi:10.1001/jamanetworkopen.2023.54577

3. clinicaltrials.gov. Prostate Cancer Detection Using the Stockholm3 Test and MR/​Fusion Biopsies (STHLM3MR-2). National Library of Medicine. Updated 2023. 2024. <https://clinicaltrials.gov/study/NCT03377881>

4. Eklund M, Jäderling F, Discacciati A, et al. MRI-Targeted or Standard Biopsy in Prostate Cancer Screening. *New England Journal of Medicine*. 2021;385(10):908-920. doi:doi:10.1056/NEJMoa2100852

5. Bratt O, Carlsson S, Fransson P, Thellenberg Karlsson C, Stranne J, Kindblom J. The Swedish national guidelines on prostate cancer, part 1: early detection, diagnostics, staging, patient support and primary management of non-metastatic disease. *Scandinavian Journal of Urology*. 2022/07/04 2022;56(4):265-273. doi:10.1080/21681805.2022.2094462

6. Epstein JI, Egevad L, Amin MB, Delahunt B, Srigley JR, Humphrey PA, the Grading C. The 2014 International Society of Urological Pathology (ISUP) Consensus Conference on Gleason Grading of Prostatic Carcinoma: Definition of Grading Patterns and Proposal for a New Grading System. *The American Journal of Surgical Pathology*. 2016;40(2)

7. Austin PC, Steyerberg EW. Events per variable (EPV) and the relative performance of different strategies for estimating the out-of-sample validity of logistic regression models. *Statistical methods in medical research*. Apr 2017;26(2):796-808. doi:10.1177/0962280214558972

8. Frank E. Harrell Jr. *Regression Modeling Strategies: With Applications to Linear Models, Logistic and Ordinal Regression, and Survival Analysis*. 2 ed. Springer Series in Statistics. Springer Cham; 2015.

9. Schoots IG, Padhani AR. Risk-adapted biopsy decision based on prostate magnetic resonance imaging and prostate-specific antigen density for enhanced biopsy avoidance in first prostate cancer diagnostic evaluation. *BJU international*. Feb 2021;127(2):175-178. doi:10.1111/bju.15277

10. Doluoğlu Ö G, Öztekin Ç V, Karabakan M, Akdemir A, Çetinkaya M. The importance of prostate volume in prostate needle biopsy. *Turk J Urol*. Jun 2013;39(2):74-7. doi:10.5152/tud.2013.019

11. Streicher J, Meyerson BL, Karivedu V, Sidana A. A review of optimal prostate biopsy: indications and techniques. *Therapeutic advances in urology*. Jan-Dec 2019;11:1756287219870074. doi:10.1177/1756287219870074

12. Cornford P, van den Bergh RCN, Briers E, et al. EAU-EANM-ESTRO-ESUR-ISUP-SIOG Guidelines on Prostate Cancer&#x2014;2024 Update. Part I: Screening, Diagnosis, and Local Treatment with Curative Intent. *European urology*. doi:10.1016/j.eururo.2024.03.027

13. Özden E, Akpınar Ç, İbiş A, Kubilay E, Erden A, Yaman Ö. Effect of lesion diameter and prostate volume on prostate cancer detection rate of magnetic resonance imaging: Transrectal-ultrasonography-guided fusion biopsies using cognitive targeting. *Turk J Urol*. Jan 2021;47(1):22-29. doi:10.5152/tud.2020.20238

14. Montesinos López OA, Montesinos López A, Crossa J. Overfitting, Model Tuning, and Evaluation of Prediction Performance. *Multivariate Statistical Machine Learning Methods for Genomic Prediction*. Springer International Publishing; 2022:109-139.

15. Fluss R, Faraggi D, Reiser B. Estimation of the Youden Index and its Associated Cutoff Point. *Biometrical Journal*. 2005;47(4):458-472. doi:<https://doi.org/10.1002/bimj.200410135>

16. Liu X. Classification accuracy and cut point selection. *Statistics in Medicine*. 2012;31(23):2676-2686. doi:<https://doi.org/10.1002/sim.4509>

**Appendix 1**

From Eklund M, Jäderling F, Discacciati A, et al. MRI-Targeted or Standard Biopsy in Prostate Cancer Screening. New England Journal of Medicine. 2021;385(10):908-920. Copyright © (2021) Massachusetts Medical Society. Reprinted with permission.


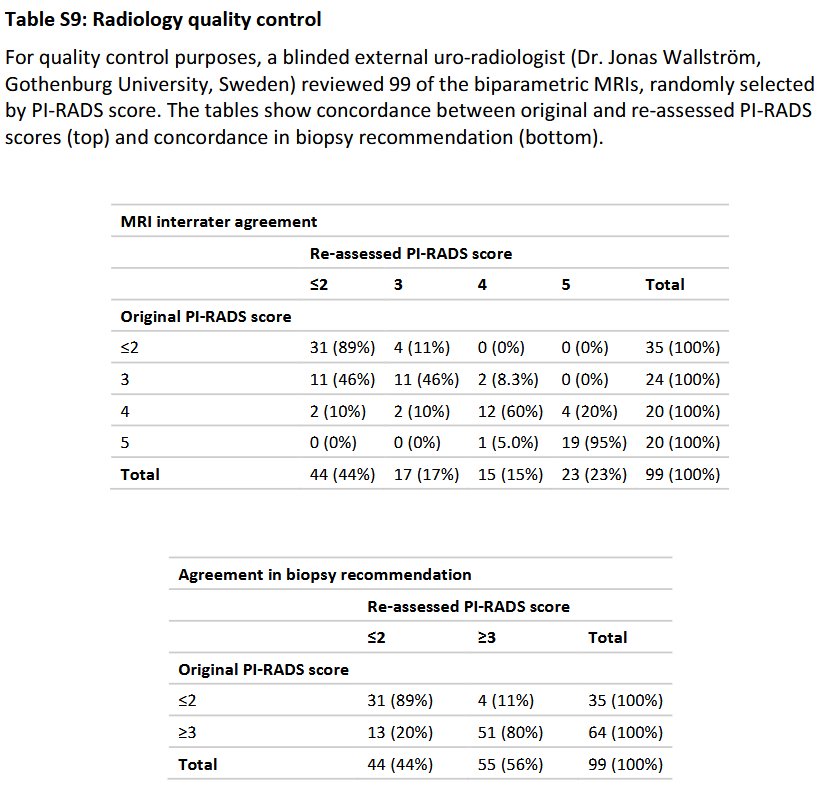

Supplement: Supplementary Document [file mmc1.docx]
