# Supplementary material for: Evaluating the performance of existing tools to predict clinically significant prostate cancer in men with indeterminate lesions on biparametric MRI and development of a novel multiplex model: a prospective cohort study
Source: eClinicalMedicine. 2025 Apr 3;82:103191. doi: 10.1016/j.eclinm.2025.103191 (PMC12002877; doi:10.1016/j.eclinm.2025.103191)
Supplement: Summary in Swedish [file mmc2.docx]

**Summary in Swedish**

Sammanfattning

Bakgrund

Obestämda lesioner på MRT av prostatan, såsom PI-RADS 3, utgör en klinisk utmaning på grund av sin tvetydiga natur, vilket försvårar biopsibeslut hos män som genomgår utredning för prostatacancer. Att förstå biomarkörers och riskkalkylatorers prediktiva kapacitet är avgörande för att förbättra det kliniska beslutsfattandet och minska antalet onödiga biopsier.

Metodik

I denna prospektiva kohortstudie inkluderades män med PI-RADS 3-fynd på biparametrisk MR (bp-MRI) som genomgick kombinerad biopsi (fusionsinriktad och systematisk) i den randomiserade kliniska studien STHLM3-MRI (första och andra omgången) och vid Capio S:t Görans sjukhus, Sverige, som representerar screening genom inbjudan, upprepad screening respektive kohorter med klinisk praxis. Datainsamlingen skedde mellan den 5 februari 2018 och den 4 mars 2020 för STHLM3-MRI-screening i första omgången, mellan den 10 november 2021 och den 20 februari 2023 för screening i andra omgången och mellan den 7 januari 2017 och den 30 juni 2023 för Capio PCC. Uppgifterna samlades in direkt från de deltagande laboratorierna med hjälp av standardiserade rapporteringsformulär, medicinska journaler och ytterligare studiespecifika datainsamlingsformulär som fylldes i av patienterna. Det primära resultatet var upptäckt av kliniskt signifikant prostatacancer (csPCa; ISUP ≥ 2) hos män med PSA ≥3ng/mL som bekräftats genom den kombinerade biopsin. Den prediktiva förmågan hos de utvärderade biomarkörerna (PSA-densitet, Stockholm3-testet, prostatavolym, volymförhållande mellan MRT-lesioner och Stockholm3-densitet) samt sju riskkalkylatorer bedömdes med hjälp av ytan under kurvan (AUC) som beräknades med logistisk regression. Sensitivitet och specificitet för att upptäcka csPCa och höggradig prostatacancer (ISUP ≥ 3) rapporterades. En fullständig fallanalys utfördes för män med fullständiga uppgifter om PSA, prostatavolym, Stockholm3-test, MRT-lesionsvolym, fynd vid digital rektalundersökning, familjehistoria med prostatacancer och tidigare biopsi. Resultaten jämfördes med analysen från den imputerade datauppsättningen.

Resultat

Av de 6 554 män som ingick i de tre kohorterna fick 1 187 PI-RADS-poäng 3 på bp-MRI och 1 146 genomgick en kombinerad biopsi. Av dem hade 900 PSA≥3ng/ml, och 656 män ingick i analysen av samtliga fall (169 från STHLM3-MRI första omgången, 72 från andra omgången och 415 från Capio PCC). Totalt sett hade 370/900 män (41%) och 258/656 män (39%) ISUP ≥2, men endast 75/900 (8%) och 50/656 (8%) hade ISUP ≥3. PSA-densitet, testade riskkalkylatorer och sannolikhetstester hade låg till måttlig AUC (intervall 0,50-0,73; PSA-densitet intervall 0,58-0,66, Stockholm3 intervall 0,59-0,67, lesionsvolymförhållande intervall 0,54-0,63) och fungerade på liknande sätt i enskilda kohorter och det kombinerade datasetet i analysen av fullständiga fall och imputerade dataset. För detektion av ISUP ≥2 baserat på STHLM3-MRI första omgången hade PSA-densitet på 0,10 en sensitivitet på 69 % (56 %, 80 %), specificitet på 49 % (39 %, 58 %) och saknade 27 % (6 %, 61 %) av ISUP ≥3, medan en PSA-densitet på 0,15 hade en sensitivitet på 37 % (25 %, 50 %), specificitet på 84 % (76 %, 90 %) och saknade 45 % (17 %, 70 %) av ISUP ≥3. Den bäst presterande modellen baserad på STHLM3-MRI inkluderade ålder, prostatavolym, Stockholm3-densitet och MR-läsionsförhållande och minskade antalet prostatabiopsier med 33% (26%, 40%) med bibehållen 98% (91%, 100%) sensitivitet för att upptäcka ISUP ≥2-cancer, specificitet på 50% (41%, 60%) och AUC på 0,82 (0,76,0,87). Samtidigt inkluderade den bäst presterande modellen baserad på det fullständiga kombinerade datasetet ålder, prostatavolym, PSA-densitet och Stockholm3-densitet och minskade prostatabiopsier med 26% (23%, 30%) med en känslighet på 90% (85%, 93%), specificitet på 36% (31%, 41%) och AUC på 0,70 (0,66, 0,74).

Tolkning

Nuvarande riskstratifieringsverktyg och enskilda biomarkörer fungerar suboptimalt för att vägleda biopsibeslut hos män med PI-RADS 3-lesioner. Resultaten belyser begränsningarna med att enbart förlita sig på PSA-densitet och understryker behovet av försiktighet i kliniska rekommendationer. Multiplexmodeller kan dock ge möjlighet att minska antalet onödiga biopsier och samtidigt bibehålla en hög känslighet för kliniskt signifikant detektion av prostatacancer. Dessa resultat bör valideras externt och utvärderas med avseende på kostnadseffektivitet.

Finansiering

Den kliniska studien STHLM3-MRI finansieras av Cancerfonden, Vetenskapsrådet, Forskningsrådet för hälsa, arbetsliv och välfärd (FORTE), Strategiska forskningsprogrammet om cancer (StratCan), Hagstrandska Minnesfonden, Region Stockholm, Svenska Druidorden, Åke Wibergs Stiftelse, Swedish e-Science Research Centre, Karolinska Institutet och Prostatacancerförbundet.
